# Synthesis of a Highly Fluorescent Quinoxalino[2,3‑*b*]quinoxaline Polycyclic Derivative via Intramolecular Michael Addition to a Squaramide Ring

**DOI:** 10.1021/acs.joc.5c03075

**Published:** 2026-02-27

**Authors:** Giacomo Picci, Jessica Milia, Vito Lippolis, Pier Carlo Ricci, Antonio Frontera, Rosa M. Gomila, Emmanuel O. Ojah, Randima D. De Silva Weerakonda Arachchige, James B. Orton, Simon J. Coles, Nathalie Busschaert, Claudia Caltagirone

**Affiliations:** † Department of Chemical and Geological Science, 3111 University of Cagliari, S.S. 554 Bivio per Sestu, 09042 Monserrato, CA, Italy; ‡ Department of Physics, University of Cagliari, S.S. 554 Bivio per Sestu, 09042 Monserrato, CA, Italy; § Department of Chemistry, 16745 Universitat de les Illes Balears, Cra. de Valldemossa, km 7.5, Palma 07122, Spain; ∥ Department of Chemistry, 5783 Tulane University, New Orleans, Louisiana 70118, United States; ⊥ UK National Crystallographic Service, School of Chemistry and Chemical Engineering, Faculty of Engineering and Physical Sciences, University of Southampton, Highfield SO17 1BJ, U.K.

## Abstract

In the presence of TBAOH, bis-indolylsquaramide (**1**) converts into the highly emissive, novel bis­(3H-pyrrolo­[1,2,3-de]­quinoxaline) exa-cyclic derivative **2**. This compound was fully characterized in solution and the solid state, with emission properties supported by DFT calculations. Derivative **2** exhibits high quantum yield in solution with aggregation-induced quenching, and a large spectral shift between solid state and solution. Reaction conditions were optimized, and a mechanism involving a double intramolecular Michael addition triggered by deprotonation, oxidation and photodecarbonylation was proposed on the basis of DFT calculations and LC-MS measurements. In addition to reporting a novel, highly π-conjugated emissive compound, this manuscript highlights an unprecedented squaramide reactivity under basic conditions, resulting in the first example of intramolecular quinoxaline moiety formation from a squaramide derivative.

## Graphical abstract

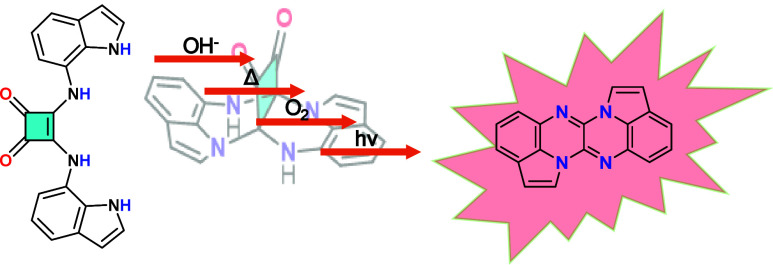

## Introduction

Supramolecular systems containing squaramides have attracted considerable attention over the past two decades due to the unique properties of the squaramide scaffold.^1^ Squaramides are well-known to act as both hydrogen-bond donors and acceptors in anion recognition,^2^ extraction with both macrocyclic^3^ and non-macrocyclic receptors,^4^ transport,^5^ and the development of supramolecular architectures for various purposes, including catalysis.^6^ The photochemical ring-opening of the cyclobutenedione core to generate 1,2-bisketenes is also well-known,^7^ and recently has been exploited to modulate anion transport in aniline-derived squaramides.^8^ Interestingly, the squaramide ring resembles a Michael acceptor, making it susceptible to nucleophilic attack, although this has not yet been reported. Squaramides have, however, been used to catalyze oxa-Michael cascade reaction as in the case of chiral 1,4-dihydropyridines^9a^ or the synthesis of enantioenriched 1,2-oxazine scaffolds,^9b^ both obtained using *Cinchona*-derived squaramides as catalysts.

Recently, we reported bis-indolylsquaramide **1** (Scheme 1) as a high affinity chloride receptor in competitive solvents, along with its transmembrane transport properties and its application in nonsteroidal anti-inflammatory drugs (NSAID) selective electrodes.^10^ When studying anion binding by receptors featuring acidic hydrogen-bond donors (e.g., squaramides), it is advisible to assess deprotonation by strong bases as the resulting electronic rearrangement often causes notable changes in the receptor’s absorption properties.^11^

**Scheme 1:**
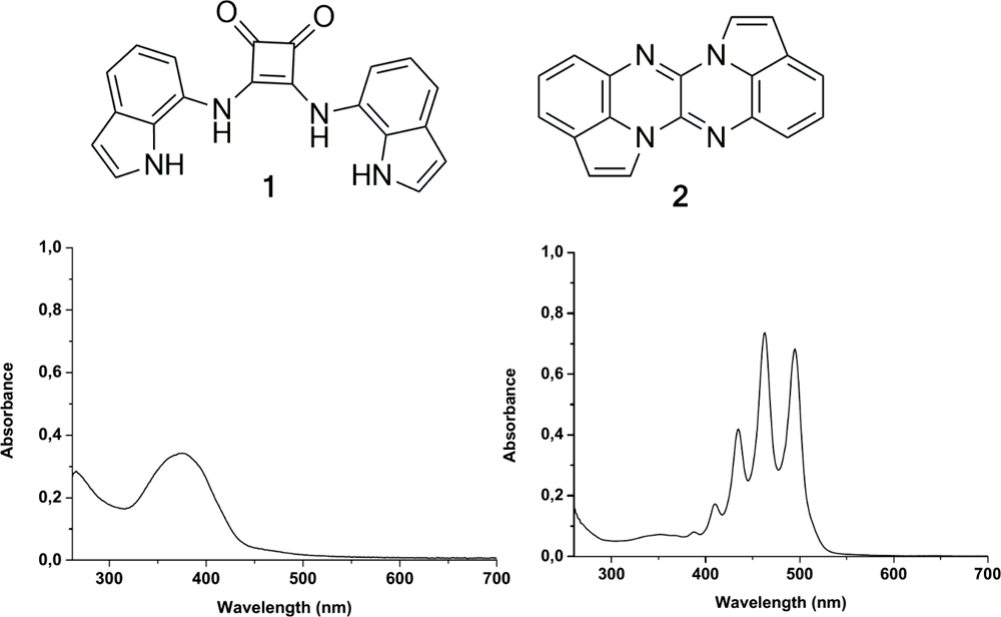
Chemical Drawings of Squaramide **1** and **2**, along with Their Corresponding UV–vis Spectra in DMSO; [**1**] = 1.5 × 10^–5^ M, [**2**] = 5.9 × 10^–5^ M

Herein, we describe an unprecedented squaramide ring reactivity under basic conditions, resulting in the first example of intramolecular quinoxaline moiety formation from a squaramide derivative.

## Results and Discussion

The addition of excess tetrabutylammonium hydroxide (TBAOH, 10 equivs) to **1** in DMSO caused a dramatic and unusual change in both its electronic absorption (Scheme 1) and emission properties. Such behavior suggested the formation of a new species in solution (Figures S1 and S2 in Supporting Information, SI), rather than the simple NH deprotonation of a squaramide derivative.^2d^ Specifically, the absorption band of **1** at 375 nm shifted to 355 nm in the presence of 10 equivs of TBAOH, and a new structured band appeared with peaks at 443, 462, and 494 nm. Concomitantly, excitation at 375 nm resulted in a structured emission band with maxima at 460, 500, and 537 nm. The optimal amount of TBAOH required to form this new species, was studied in detail. Time-dependent monitoring of the new absorption and emission bands (see text in (SI) and Figures S3 and S4) showed that the best results were obtained in DMSO with 10 equivs of TBAOH.

To isolate the new fluorescent species observed in solution, squaramide **1** was reacted with 10 equivs of TBAOH (1 M in MeOH) in 1,4-dioxane. The choice of the solvent was dictated by the ease in the reaction workup. The fluorescent product was isolated as a dark orange solid in 73% yield after column chromatography (see SI for details and Figures S5 and S6). The formation of **2** under various conditions was monitored by HPLC-MS (see below). The reaction was performed using various bases (TBAOH, DBU, and triethylamine) and various TBA salts (TBAF, TBACl, TBABr, TBAI, TBANO_3_, TBAH_2_PO_4_, TBA_2_SO_4_, and TBAHCO_3_). In all cases, the reaction was carried out at 80 °C under ambient atmosphere with 10 equivs of base/salt in DMSO. Notably, as further described below, the formation of **2** was observed also in the presence of TBAF and, at lower extent, DBU, as expected from the p*K*_a_ values of their conjugated acids in DMSO.^12^ In this context all the following discussion will be conducted considering TBAOH as the base.

Crystals suitable for single-crystal X-ray diffraction analysis were obtained by slow evaporation of a THF solution (see SI, Figure S7 and Table S1). The structure revealed the formation of the novel compound 3*H*-pyrrolo­[1,2,3-*de*]­3*H*-pyrrolo­[3′,2′,1′:8,1] quinoxalino­[2,3-*b*]­quinoxaline (**2**, Scheme 1), consisting of two fused 3H-pyrrolo­[1,2,3-de]­quinoxaline units. Its structure shows a perfectly planar molecule, which can also be considered as consisting of two pyrrole rings fused to a quinoxalino­[2,3-*b*]­quinoxaline heteroacene core, and lying on a crystallographic inversion center at the midpoint of the C1–C1^i^ bond (^i^ = −x, 1–y, 1–z; Figure 1). In the crystal packing, molecules of **2** are slip-stacked into columns along the *a*-axis, with intermolecular distances of 3.4266 (10) Å and intercentroid distances of 4.7982 (2) Å.

**Figure 1:**
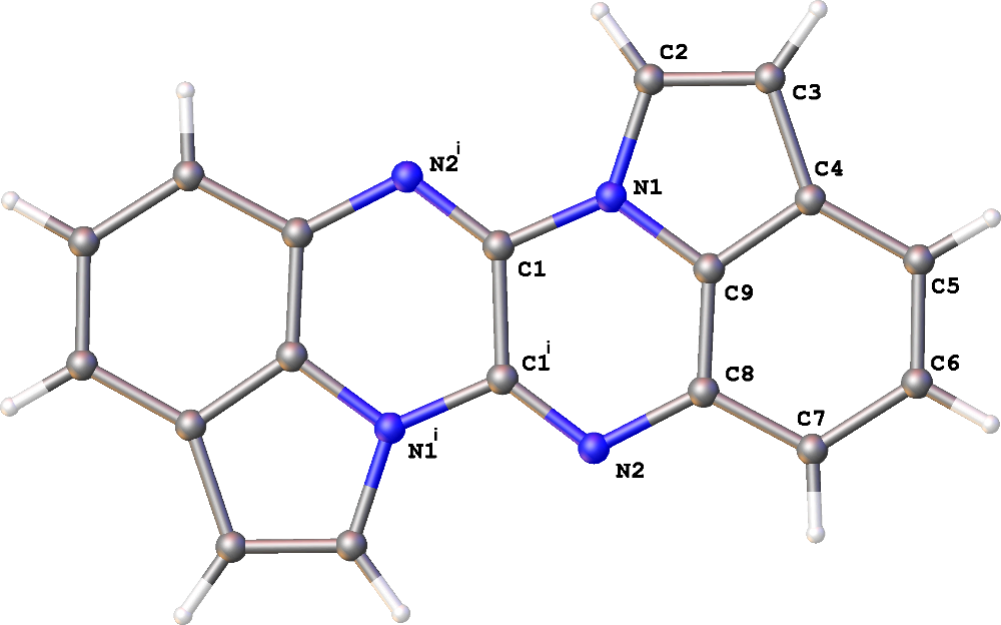
ORTEP view of compound **2** with the adopted atom labeling scheme. Thermal ellipsoids are drawn at 50% probability level; ^i^ = −x, 1–y, 1–z.

Each column is surrounded by four symmetry-related columns containing molecules of **2** oriented perpendicular to those in the central column (Figure 2). In this way, alternating pinwheel-like arrangements of columns of **2** are formed along the *a*-direction.

**Figure 2:**
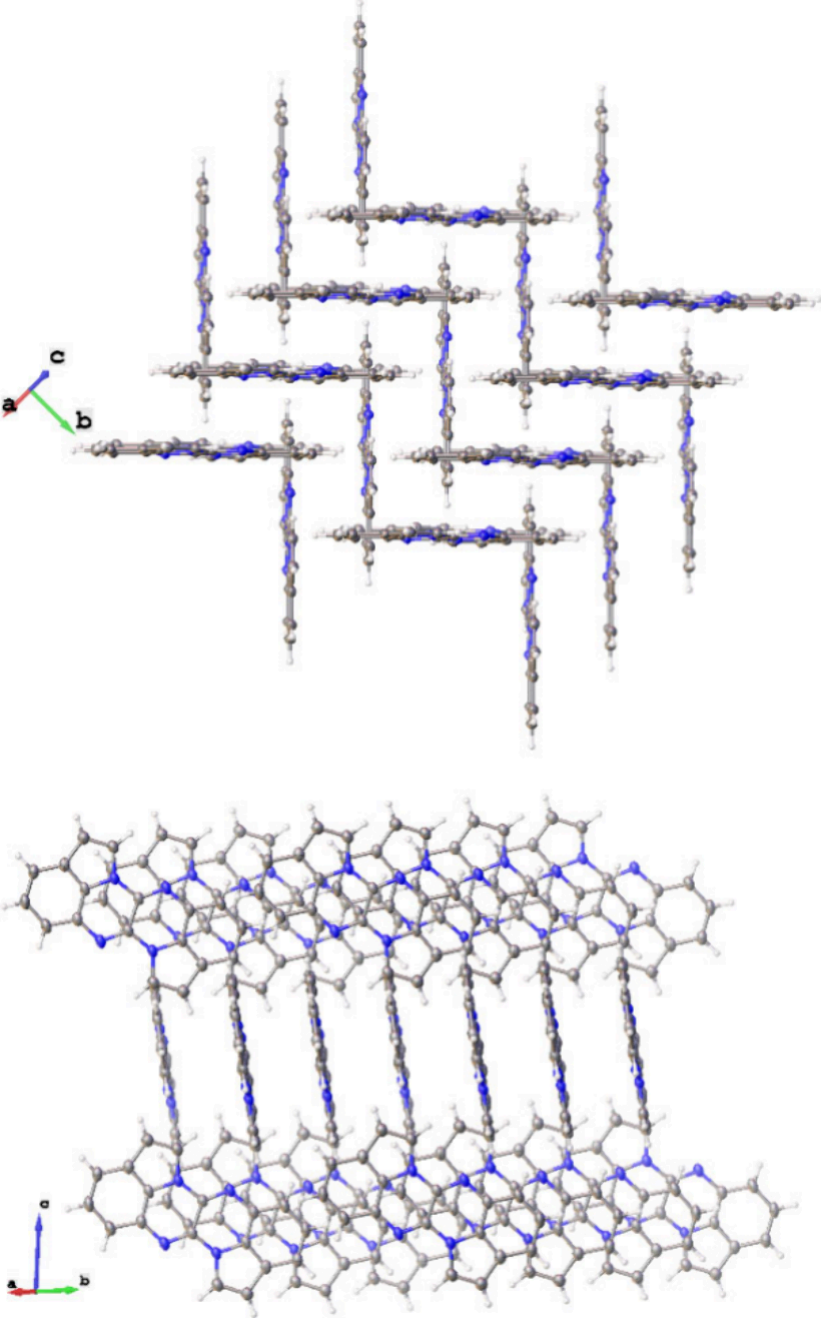
Alternative views of mutually perpendicular assemblies of molecules of **2**, arranged in slip-stacked columns along the a-direction.

Only few examples of condensed polyaromatic systems formed by fusion of an electron-poor quinoxaline and electron-rich pyrrole — such as indolizino­[5,6-*b*]­quinoxaline,^13^ pyrrole­[1,2-*a*]­quinoxalines,^14^ pyrrole­[2,3-*b*]­quinoxalines,^15^ and pyrrole­[3,4-*b*]­quinoxalines^16^ — have been reported. A synthetic protocol for functionalized 3*H*-pyrrolo-[1,2,3-de] quinoxalines has only recently been described.^17^ Some of these compounds exhibit interesting optical properties for optoelectronic applications,^13^ show good photostability, photosensitizing ability, and bioimaging applicability,^18^ and have also been used for the development of porous materials,^19^ or as organic field-effect transistors (OFTEs).^20^ However, to the best of our knowledge, no heteroacene compound similar to **2**, i.e. featuring two pyrrole rings fused to a quinoxalino­[2,3-*b*]­quinoxaline heteroacene core, has been previously reported. Given the extended π-conjugation, we expected **2** could display intriguing optical properties.

Preliminary density functional theory (DFT) calculations (see SI for details), suggest that all condensed rings in **2** contribute almost equally to the HOMO and LUMO. The small HOMO–LUMO gap (2.86 eV) is consistent with the compound’s color. The terminal benzo-pyrrole units contribute more to the HOMO–1 than the central fused pyrazine rings (Figure 3), while in the LUMO+1, the pyrrole rings contribute less than the four fused six-membered rings of the quinoxalino­[2,3-*b*]­quinoxaline heterocene system.

**Figure 3:**
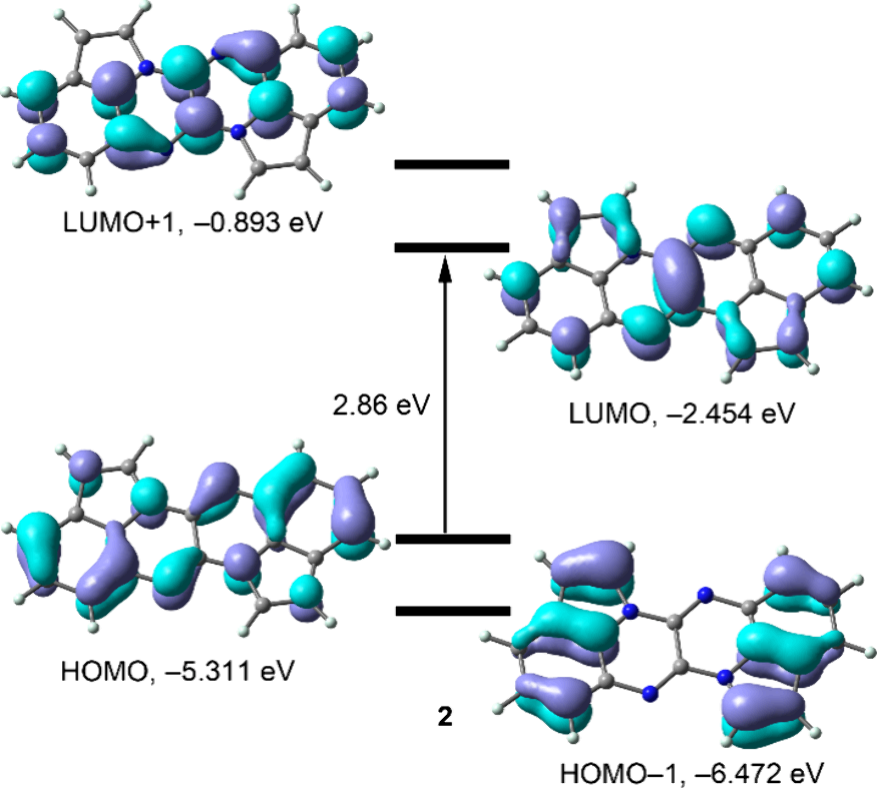
Frontier Molecular Orbitals (MOs) of **2** calculated at the B3LYP-D4/def2-TZVP level of theory.

We first considered the optical properties of **2** in the solid state. The absorption spectrum shows bands at 432, 461, and 495 nm, similar to those observed for squaramide **1** in DMSO upon addition of 10 equivs of TBAOH, along with an additional band at 540 nm (Figure S8).

Time-dependent DFT (TD-DFT) calculations support assigning the band at 540 nm as a fingerprint of compound **2** formation. Specifically, the S_0_ → S_2_ transition, calculated at 543.5 nm with an oscillator strength of *f* = 0.0146, is in excellent agreement with the experimental data. This excitation is composed of 60% HOMO → LUMO+1 and 40% HOMO–1 → LUMO contributions.

A 3-D contour plot (Figure 4A) shows a broad excitation region from 250 to 540 nm, with clear maxima near 460, 500, and most prominently at 540 nm. The emission spectrum is largely independent from the excitation wavelength, consistently showing a broad band from 600 to 800 nm with a maximum at 640 nm (Figure S8B). A distinct shoulder at lower energies suggests multiple emissive components.

**Figure 4:**
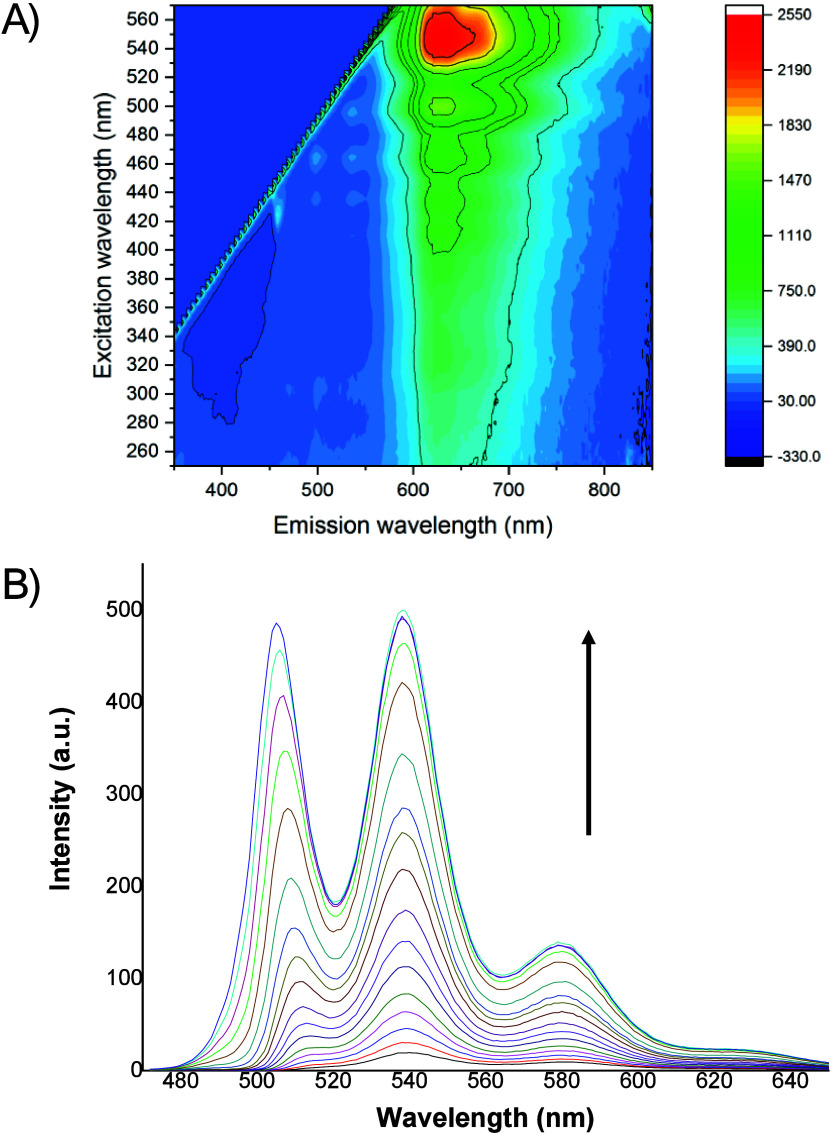
A) 3-D contour plot of **2**; B) Changes in the emission spectra of **2** in DMSO upon dilution from 1.67 × 10^–3^ to 5.89 × 10^–5^ M (λ_exc_ = 375 nm).

Time-resolved luminescence at each excitation maximum confirms that the emission wavelength remains nearly unchanged, while notable differences appear in the temporal domain (Figure S9). Radiative recombination thus involves distinct excited states with different decay dynamics: higher-energy emissions decay much faster than lower-energy and near-infrared ones, which show minimal lifetime variations. Emission at 570 nm requires a biexponential model, with a fast component nearly coinciding with the excitation pulse—suggesting parallel nonradiative pathways. In contrast, emissions at 650–670 nm fit a single-exponential decay (τ = 1.9 ns), consistent with a single recombination mechanism.

To investigate the optical properties of **2** in solution, crystals of **2** were dissolved in DMSO (1.67 × 10^–3^ M) and the solution was diluted to a final concentration of 5.89 × 10^–5^ M. Dilution resulted in a 500-fold increase in raw emission intensity (Figure 4B), suggesting aggregation-caused quenching (ACQ).^21^ Dilution caused a red shift in the emission bands, with maxima at 505, 539, and 580 nm, and a shoulder at 628 nm. Relative quantum yields (Φ) were determined in various solvents. Remarkably high values (Φ ≥ 0.85) were obtained in both aprotic and protic solvents (Table S2). Notably, **2** exhibits solvatochromism (Figure S10).

The fluorescence changes in the solid state and in solution were further investigated using TD-DFT calculations. To simulate the fluorescence in solution, a single molecule of **2** was modeled with solvent effects via a polarizable continuum model. For the solid-state, a slipped π-stacked dimer was employed to simulate the 1D columnar arrangement observed in the crystal. The dimer exhibits a broad emission band from 600 to 900 nm (Figure S11) with a distinct maximum at 690 nm, in reasonable agreement with the experimental data. Although the emission intensity is low, normalized intensities were used to facilitate visualization and comparison. This low intensity suggests that π-stacking in the solid-state leads to significant fluorescence quenching. Indeed, the relative quantum yield of compound **2** in the solid state is notably low (0.03).

In contrast, the monomeric model in solution exhibits significantly higher emission intensity, with three well-defined emission bands at shorter wavelengths. Solvatochromic behavior was also investigated computationally in DMSO, acetonitrile, and hexane. Calculations predict a significant red shift (ca. 70 nm) in hexane compared to DMSO, consistent with experimental observations. Since the simulations employed a dielectric continuum model (CPCM), the observed shifts are attributed primarily to differences in bulk solvent polarity. Consequently, the computed spectrum for acetonitrile closely resembles that for DMSO, which deviates from the experimental observations. This discrepancy highlights the limitations of implicit solvation models, which do not account for specific solute–solvent interactions within the first solvation sphere (e.g., hydrogen bonding or directional coordination) or aggregation phenomena that may vary between solvents. Furthermore, while the dimer model used for solid-state calculations captures the essential features of π-stacking interactions, it inevitably oversimplifies long-range packing effects and extended intermolecular coupling present in the crystal lattice.

The unexpected formation of **2** from **1** led us to investigate the underlying mechanism. The requirement for TBAOH suggests a deprotonation step, while the electrophilic nature of the squaramide core suggests Michael addition reactions. A plausible reaction mechanism (Scheme 2), supported by DFT calculations (Figures S12 and S13), involves an initial acid–base reaction, in which the negative charge on the deprotonated squaramide N atom is stabilized through a strong hydrogen bond with an indole NH group in intermediate INT1 (Scheme 2). This pathway also requires a single water molecule (from the reaction environment) to be involved. The anionic squaramide N atom abstracts a proton from the interacting indole NH group, whose nitrogen atom then attacks a carbon atom of the four-membered ring via a Michael addition through transition state TS1, forming intermediate INT2. The proton transfer during the subsequent Michael addition of the second indole nitrogen atom is facilitated by the water molecule, forming compound **A** in its enol form. After a rapid equilibrium shift to the keto form, **A** undergoes oxidation, to yield the diketone ring **B**. The final step in the formation of **2**, requiring light, is likely a photodecarbonylation. Previous studies showed that photodecarbonylation of α-diketones is a rapid process involving triplet state species,^22^ and consequently has not been studied further herein.

**Scheme 2:**
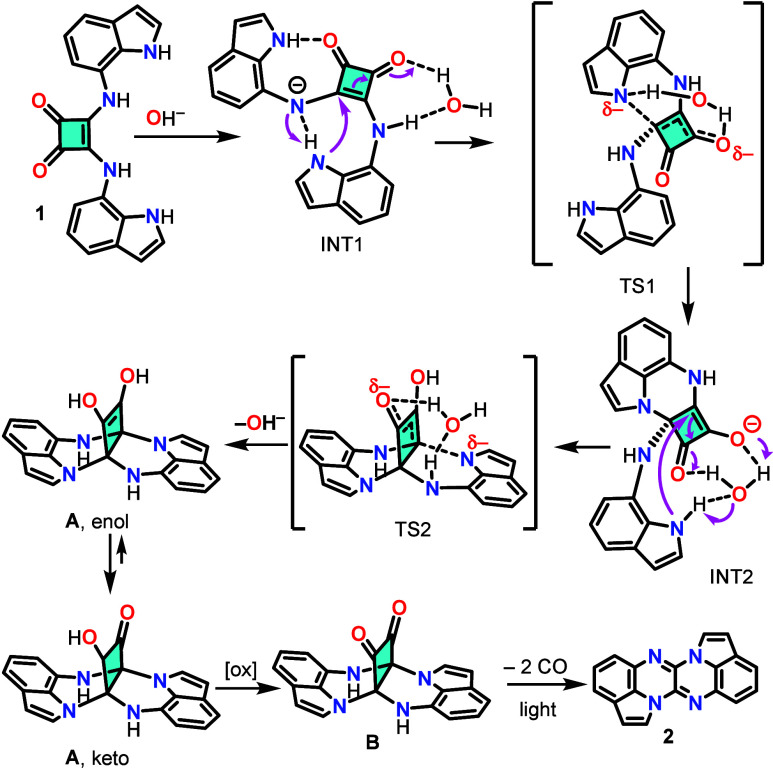
Proposed Mechanism for the Formation of **2** from **1** under Basic Conditions

DFT calculations (see SI) were performed to support the mechanism proposed. Initially, the energy profile for the formation of compound **A** (enol form), was analyzed (Figure S12). The initial acid–base reaction is highly favorable, leading to the formation of INT1. The energy barrier for the first Michael addition, yielding INT2, is 19.3 kcal/mol. The second Michael addition to afford **A**, with a barrier of 25.5 kcal/mol, represents the rate-determining step, and would require heating for the reaction to proceed. The enol form of **A** is only 3.2 kcal/mol more stable than the starting materials and significantly less stable than INT1, making INT1 the thermodynamically favored species. It is important to highlight that, in the absence of a water molecule in the calculations, the barrier for the second Michael addition increases to 80.5 kcal/mol, thus underscoring the crucial role of the solvent molecule in facilitating the process. A detailed discussion on the energy profile for the transformation of compound **A** (enol form) into **2** is reported in the SI (Figure S13).

The formation of **2** under various conditions was monitored by HPLC-MS to support the proposed mechanism. Only in the presence of strong bases like TBAOH and DBU (Figures S14–S29) did **2** form in significant amount, whereas weaker bases like triethylamine and other TBA salts did not lead to the formation of **2**. Among these, only TBAF was also able to facilitate the conversion of **1** into **2** (). In fact, fluoride often induces deprotonation of hydrogen-bond donors through the formation of HF_2_^–^.^23^ The second part of the mechanism involves a double Michael addition on the squaramide ring. DFT calculations indicate that the second addition has a high activation energy, necessitating heating to proceed efficiently. Indeed, when the reaction was performed with 10 equivs of TBAOH in DMSO at room temperature (∼25 °C), degradation of squaramide **1** was observed but no formation of product **2** (Figure S27). The next stage of the proposed mechanism involves keto–enol tautomerization, followed by oxidation to form an α-diketone. To minimize the presence of O_2_, the reaction was performed under argon. However, conversion to **2** was not completely inhibited under these conditions (Figure S28), the reaction likely still enabled by DMSO acting as a mild oxidant. This also explains why dioxane (a peroxide-former) and DMSO were the most effective solvents for forming **2** in the initial screening experiments. The final step is the photodecarbonylation of the α-diketone and, as expected, the formation of **2** did not occur in the dark (Figure S29). All the reaction condition tested are summarized in Figure 5.

**Figure 5:**
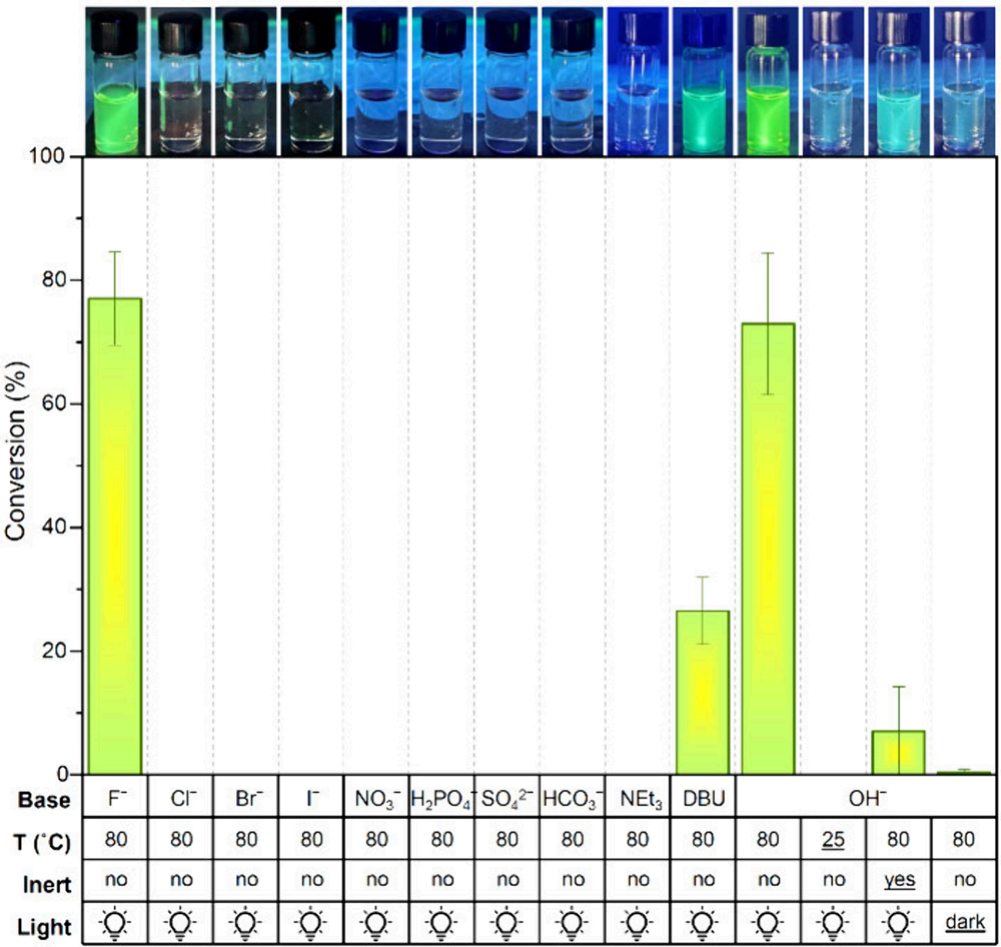
Conversion of squaramide **1** to product **2** after 2.5 h in DMSO in the presence of 10 equivs base (anionic bases were used as TBA salts). All results are the average of 3 independent repeats. Photographs are the reaction mixture diluted in MeCN irradiated with a 365 nm UV lamp.

## Conclusions

In conclusion, this study reports on the first example of intramolecular Michael additions to a squaramide ring with the one-pot formation of the exa-cyclic derivative **2** from bis-indolylsquaramide **1**. It also highlights the critical roles that heat, light, a strong base, and oxygen play, as investigated by HPLC-MS and supported by DFT calculations. The study underscores the essential role of water in facilitating key steps like the Michael addition. This unprecedented rearrangement of the squaramide ring offers new insights into the synthetic potential of squaramide derivatives. Notably, the highly π-conjugated derivative **2** is the first example of a novel quinoxalino­[2,3-*b*]­quinoxaline polycyclic system, with promising optoelectronic applications and tunable optical properties through further functionalization. Ongoing studies are underway in our laboratories.

## Supplementary Material





## Data Availability

The data underlying this study are available in the published article and its .

## Notes

FAIR data, including the primary NMR FID files, for compounds **2** (ZIP)
Supporting Information includes materials and methods, details on the synthesis and characterization of compound **2**, as well as details on the photophysical characterization both in solution and in the solid states, details on LC-MS studies, crystallographic and DFT calculation data (PDF)
