# Supplementary figures and images for: Synthesis of a Highly Fluorescent Quinoxalino[2,3‑b]quinoxaline Polycyclic Derivative via Intramolecular Michael Addition to a Squaramide Ring
Source: J Org Chem. 2026 Feb 27;91(10):3751–8. doi: 10.1021/acs.joc.5c03075 (PMC12993856; doi:10.1021/acs.joc.5c03075)

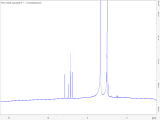

Supplement: Supplementary file 1 [file jo5c03075_si_001.zip › NMR FID/1/pdata/1/thumb.png]

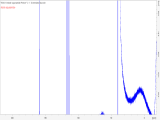

Supplement: Supplementary file 1 [file jo5c03075_si_001.zip › NMR FID/2/pdata/1/thumb.png]
