# Supplementary material for: Synthesis of a Highly Fluorescent Quinoxalino[2,3‑b]quinoxaline Polycyclic Derivative via Intramolecular Michael Addition to a Squaramide Ring
Source: J Org Chem. 2026 Feb 27;91(10):3751–8. doi: 10.1021/acs.joc.5c03075 (PMC12993856; doi:10.1021/acs.joc.5c03075)
Supplement: Supplementary file 2 [file jo5c03075_si_002.pdf]

Synthesis of a Highly Fluorescent Quinoxalino[2,3-b]quinoxaline polycyclic derivative via Intramolecular Michael Addition to a Squaramide Ring

Giacomo Picci, Jessica Milia, Vito Lippolis, Pier Carlo Ricci, Antonio Frontera, Rosa M. Gomila, Emmanuel O. Ojah, Randima D. De Silva Weerakonda Arachchige, James B. Orton, Simon J. Coles, Nathalie Busschaert,* Claudia Caltagirone*

S1. Materials and methods page S2

S2. Solution studies on squaramide 1 page S3

S3. Synthesis of *pyrrolo[1,2,3-de]pyrrolo[3',2',1':8,1]quinoxalino[2,3-b]quinoxaline* 2 page S5

S4. Crystallographic data page S8

S5. Solid state luminescence studies page S10

S6. Solution luminescence studies page S11

S7. Theoretical calculations of the reaction mechanism page S13

S8. Cartesian coordinates and thermodynamic data page S15

S9. LC-MS studies of the reaction mechanism page S21

S1. Materials and methods

All solvents and starting materials were purchased from commercial sources where available. Squaramide **1** was synthesised using a literature procedure.[1] UV-Vis spectra were obtained using an Agilent Cary 60 Spectrophotometer. Emission spectra in solution were obtained using an Agilent Cary Eclipse spectrofluorometer. Relative quantum yields in solution were obtained using fluorescein in 0.1 M NaOH as a reference. Relative quantum yields in the solid state were obtained using Ce:YAG as a reference. NMR spectra were obtained using a Bruker Avance Neo 600 with cryoprobe. ^{13}C NMR spectra were collected proton decoupled. NMR visualization and processing was carried out using MestReNova 15.0. Chemical shifts (δ) are reported in parts per million (ppm) and calibrated to the residual solvent peak in DMSO-d_6 (δ = 2.50 (^{1}H) and 39.52 ppm (^{13}C)). Coupling constants (*J*) are given in Hertz (Hz). The following abbreviations are used for spin multiplicity: d = doublet, dd = doublet of doublets, m = multiplet. LC-MS data was collected on a Thermo Fisher Scientific Vanquish Flex UHPLC with variable wavelength detector and ISQ EC mass spectrometer, using a Hypersil GOLD C18 column.

Three-dimensional fluorescence (3D-FL) maps were acquired using a Jasco FP-8550ST spectrofluorometer equipped with a 450 W xenon lamp (Horiba Ltd., Kyoto, Japan) as the excitation source. Measurements were performed in very-low sensitivity mode over an excitation range of 300–500 nm, with a fixed spectral bandwidth of 5 nm for excitation. Optical quantum yield (QY) measurements were carried out with the same spectrofluorometer coupled to an integrating sphere, using a 2 mm-thick sample holder and an excitation wavelength of 540 nm. A mono-crystalline Ce:YAG standard (Scientific Materials, USA) was used as the reference for relative QY determination at 460 nm. Emission spectra were recorded in emission mode, with both excitation and emission bandwidths set to 2.5 nm, and a detector response time of 0.2 s.

Time-resolved photoluminescence (TRPL) decay profiles were obtained by exciting the samples with an optical parametric amplifier (Light Conversion TOPAS-C) pumped by a regenerative Ti:sapphire amplifier (Coherent Libra-HE, Coherent Inc., Santa Clara, CA, USA) operating at 1 kHz repetition rate. The photoluminescence signal was detected using a streak camera (Hamamatsu C10910, Hamamatsu Photonics, Hamamatsu City, Japan).

[1] Picci, G.; Kubicki, M.; Garau, A.; Lippolis, V.; Mocci, R.; Porcheddu, A.; Quesada, R.; Ricci, P. C.; Scorciapino, M. A.; Caltagirone, C., *Chemical Communications* **2020,** *56* (75), 11066-11069;

S2. Solution studies on squaramide 1

A detailed study of the behaviour of squaramide **1** in the presence of increasing amount of TBAOH (1 M in MeOH) was conducted in DMSO. The UV-Vis spectrum of squaramide **1** shows a characteristic band centred at 375 nm (ε = 23000 cm^{-1} M^{-1}) whereas no emission was observed (Figure S1).

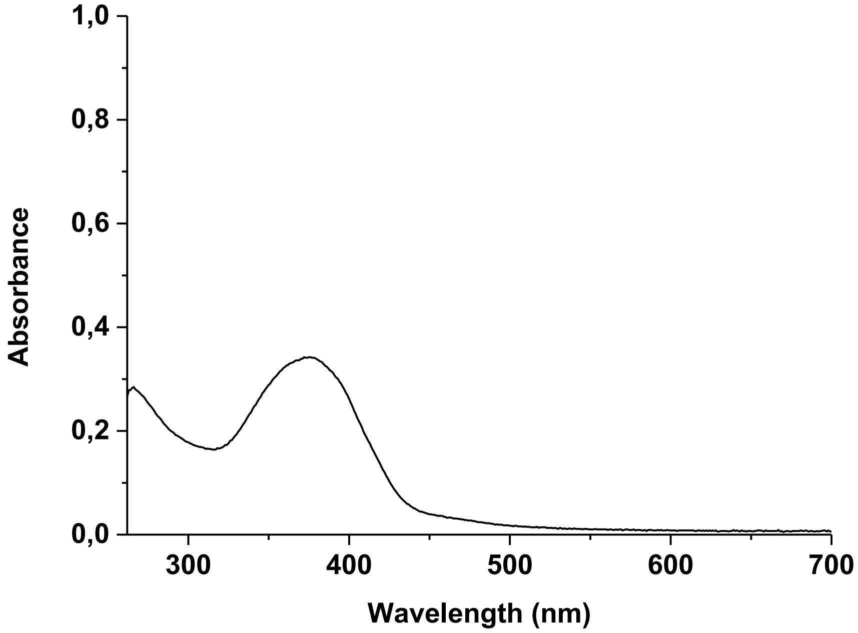

Figure S1. UV-Vis spectrum of squaramide **1** (($1.5x10^{-5}$ M) in DMSO.

I

nterestingly, upon the addition of 10 equivs. of TBAOH, the UV-Vis spectrum of squaramide **1** undergoes a hypsochromic shift of 20 nm (from 375 to 355 nm) with the concomitant enhancement of the absorbance, accompanied by the appearance of three smaller bands at 443 nm, 462 nm, and 494 nm (Figure S2a). Moreover, upon excitation at 375 nm, a structured emission band with maxima at 474 nm, 501 nm, 535nm, and 564 nm was observed (Figure S2b).

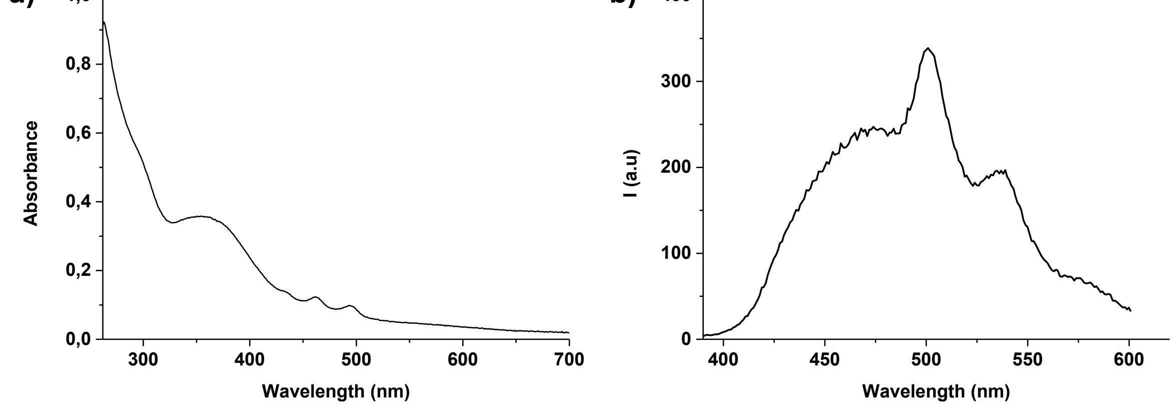

Figure S2. a) UV-Vis spectrum and b) fluorescence spectrum of squaramide **1** ($1.5x10^{-5}$ M) in DMSO upon the addition of 10 equivs of TBAOH 1M in MeOH.

To determine the precise amount of base and the reaction time required for the formation of the new species, the UV-Vis (Figure S3) and emission spectra (Figure S4) of the squaramide **1** in DMSO were recorded following the addition of 1, 3, 8, and 10 equivs. of TBAOH at various time points (0, 5, 10, 30 mins, and 24 hours). Although the new species started to form in the presence of 3 equivs of TBAOH after 24 h (figure S3C and S4C), higher amount of base (e.g., 5 or 10) is needed to obtain it with higher yields and in shorter reaction times. However, as reported in Figure S4d, the best performance was observed in the presence of 10 equivs. of TBAOH, where the maximum absorbance was reached within 10 minutes, and remained constant up to 24 hours.

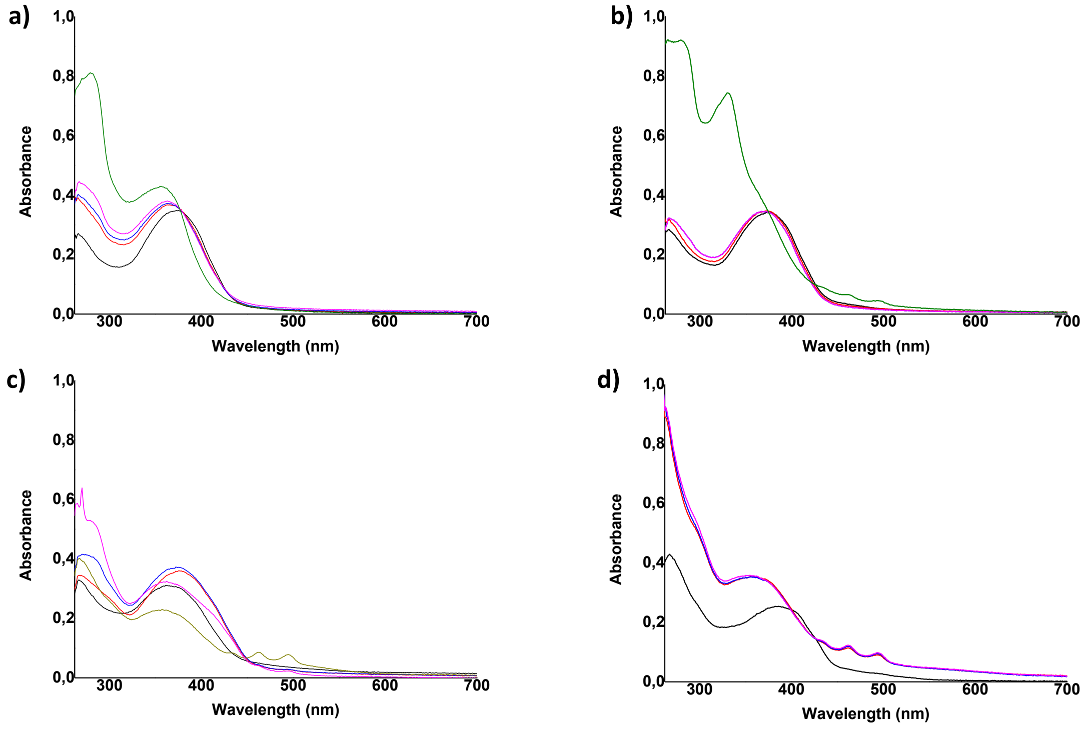

Figure S3. Absorption spectra in DMSO of a solution of **1** ($1.5x10^{-5}$ M in DMSO) upon the addition of 1 (a), 3 (b), 8 (c), 10 (d) equivs. of TBAOH ($5x10^{-3}$ M DMSO) at different reaction times (t_0 = 0 min, t_1 = 5 min, t_2 = 10 min, t_3 = 30 min, and t_4 = 24 h). Black = t_0, Red = t_1, Blue = t_2, Pink = t_3, Green = t_4.

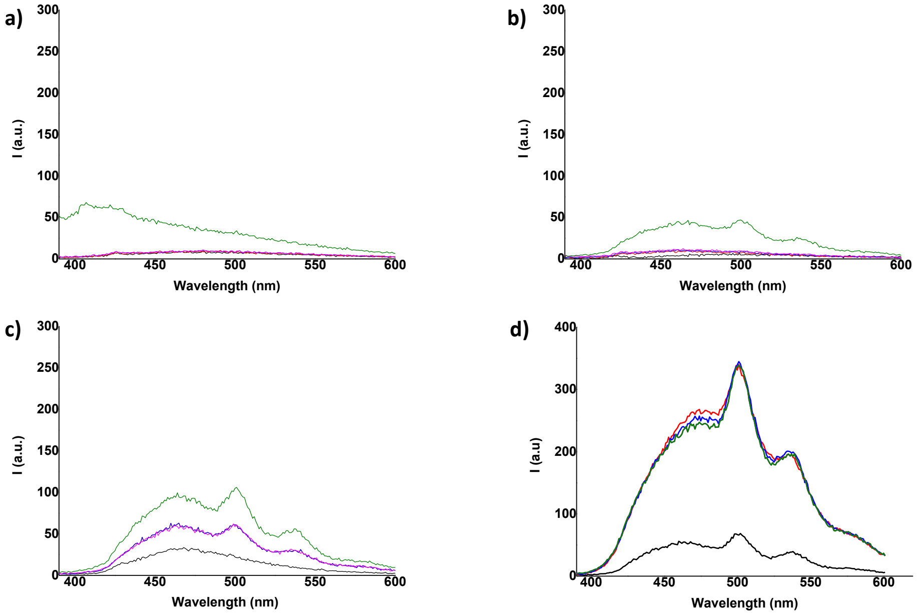

Figure S4. Emission spectra in DMSO of a solution of **1** ($1.5x10^{-5}$ M in DMSO) upon the addition of 1 (a), 3 (b), 8 (c), 10 (d) equivs. of TBAOH ($5x10^{-3}$ M DMSO) at different reaction times (t_0 = 0 min, t_1 = 5 min, t_2 = 10 min, t_3 = 30 min, and t_4 = 24 h). Black = t_0, Red = t_1, Blue = t_2, Pink = t_3, Green = t_4.

S3. Synthesis of *pyrrolo[1,2,3-de]pyrrolo[3',2',1':8,1]quinoxalino[2,3-b]quinoxaline]* 2

A solution of TBAOH (1 M in MeOH, 2.5 g, 3 mmol) was added dropwise to a solution of **1** (100 mg, 0.3 mmol) in 1,4-dioxane (20 mL, the resulting concentration of **1** in the mixture was $1.5 \cdot 10^{-2}$ M). The mixture was stirred for 16 h and heated at 80°C with an oil bath. The progress of the reaction was checked by thin layer chromatography (TLC) in EtAcO:Hexane 1:1 (v/v) following the appearance of a fluorescent spot. After completion, the solvent was removed under reduced pressure. The residue was dissolved in CH_2Cl_2 (150 mL) and washed with water (15 mL) to remove residual TBAOH. The organic phase was dried over anhydrous Na_2SO_4, filtered, and the solvent was removed under reduced pressure. The resulting oil was purified by flash column chromatography (EtAcO:Hexane 1:1 (v/v), RT: 0.8) affording the product as a dark orange solid. Yield: 73 % (56.5 mg). Melting point: > 200 °C; MS (ESI) m/z: $[M+H]^+$ calcd. for $C_{18}H_{11}N_4$ 283.1; found 283.1; ^{1}H NMR (600 MHz, DMSO-d_6) δ 7.97 (d, J = 3.4 Hz, 2H), 7.46 (dd, J = 6.4, 2.1 Hz, 2H), 7.26 – 7.11 (m, 4H), 6.95 (d, J = 3.4 Hz, 2H); ^{13}C{1H} NMR (151 MHz, DMSO-d_6 with drop of trifluoroacetic acid) δ 142.2, 131.6, 127.6, 127.2, 125.2, 121.7, 120.9, 119.6, 110.9. Elemental Analysis: Anal. Calcd for $C_{18}H_{11}N_4$: C, 76.58; H, 3.57; N, 19.85. Found: C, 76.49; H, 3.55; N, 19.81

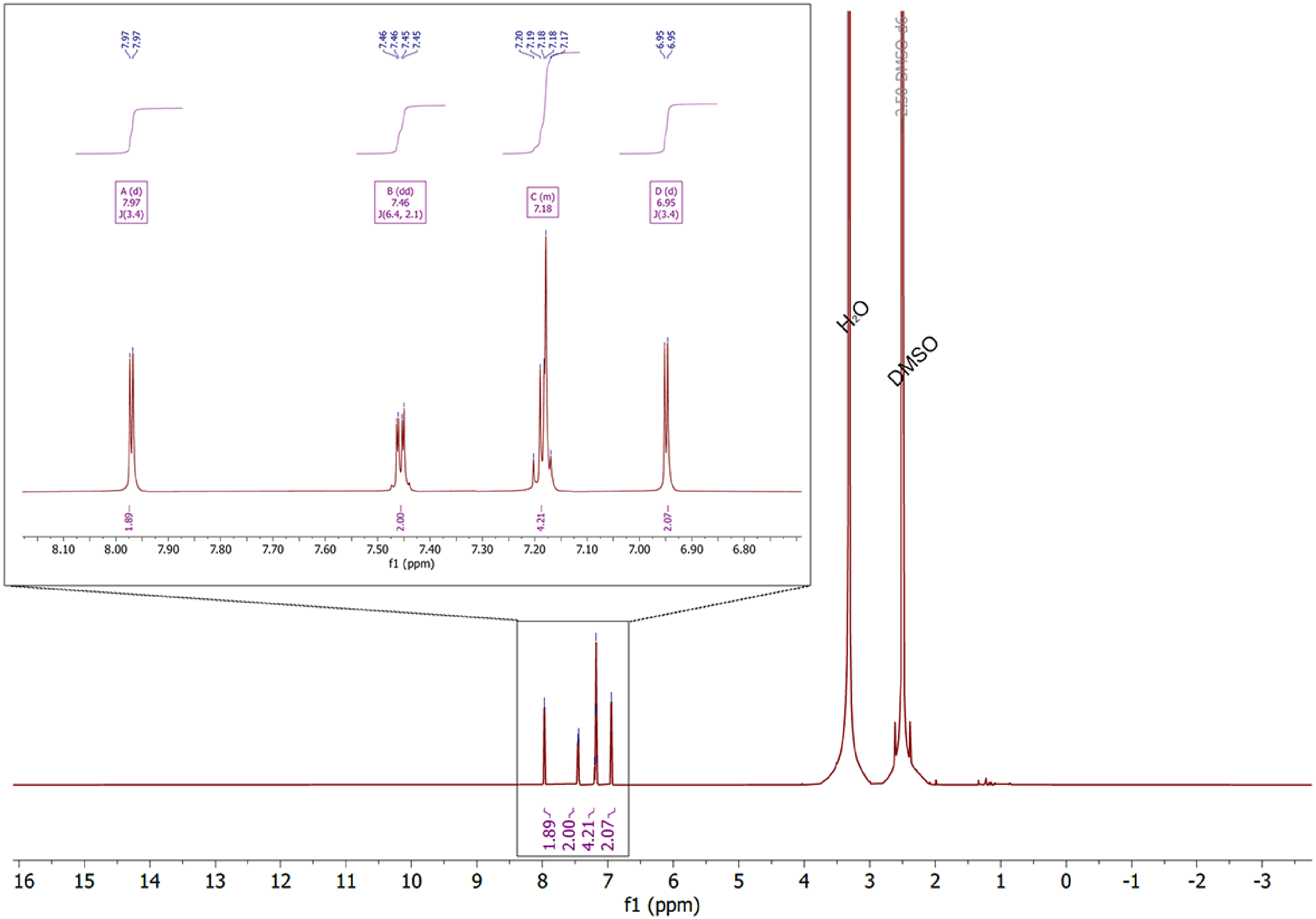

Figure S5. ^{1}H NMR spectrum (600 MHz) of **2** in DMSO-d_6 at 298 K at maximum solubility.

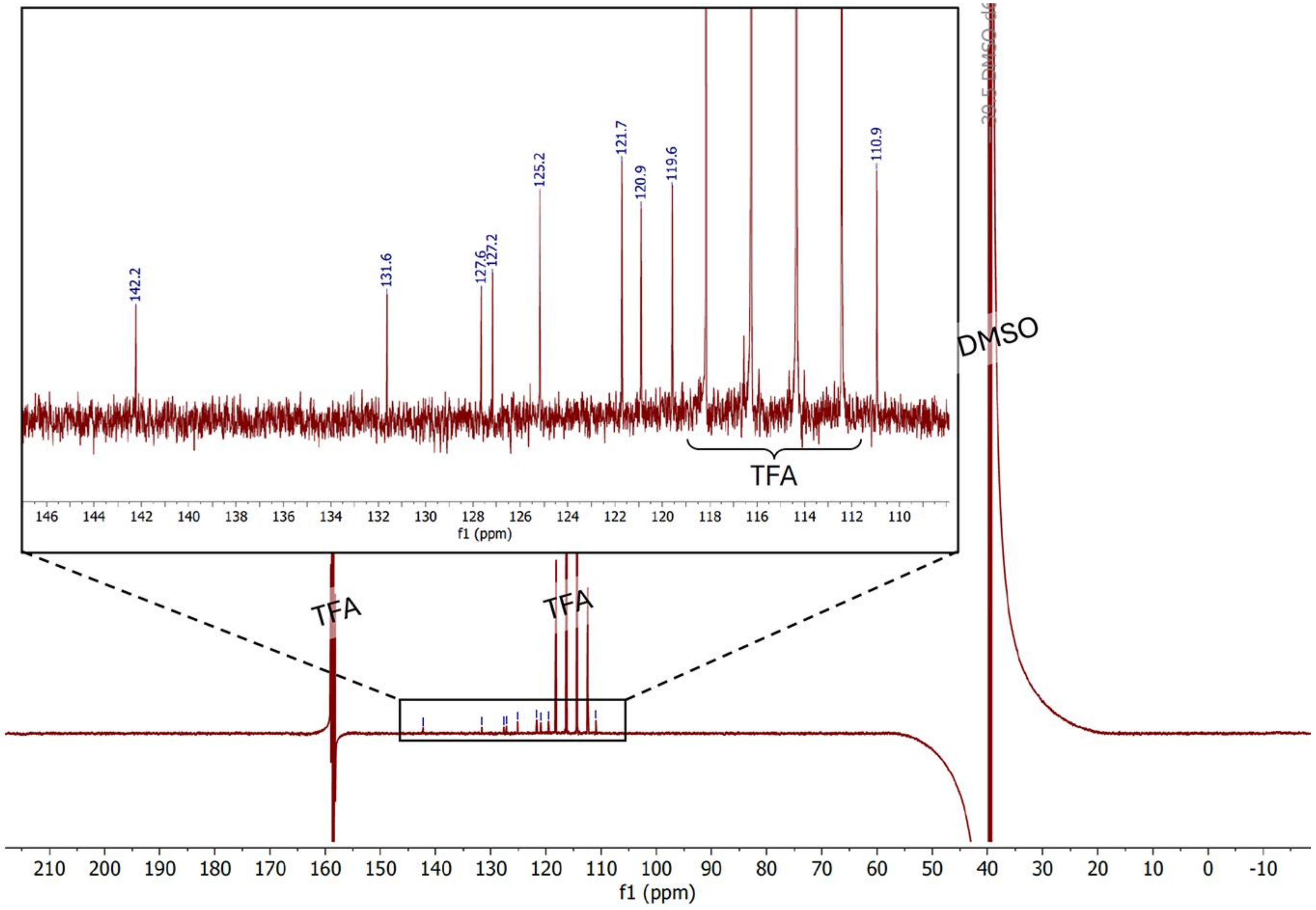

Figure S6. ^{13}C NMR spectrum (150 MHz) of **2** in DMSO-d_6 at 298 K at maximum solubility. A drop of TFA was needed to increase solubility and observe the peaks.

S4. Crystallographic data

A suitable crystal of compound **2**, with dimensions 0.26 × 0.06 × 0.02 mm^3, was selected from the dark orange needle-shaped crystals obtained by recrystallised from THF. The crystal was mounted on a MITIGEN holder using perfluoroether oil and analysed on a Rigaku 007VHF diffractometer, equipped with Varimax confocal mirrors, a UG2 Universal goniometer, and HyPix 6000 detector and an Oxford Cryosystems low temperature device. The crystal was kept at a steady T = 100(2) K during data collection. The structure was solved using the **ShelXT** 2018/2 (Sheldrick, 2018) solution program via dual-space methods, with **Olex2** 1.5 (Dolomanov *et al.*, 2009) as the graphical interface. The model was refined using **ShelXL** 2018/3 (Sheldrick, 2015) with full-matrix least squares minimisation on $\boldsymbol{F}^2$.

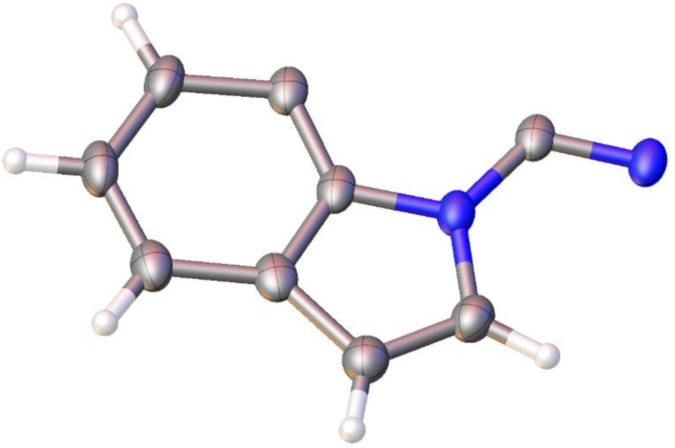

Figure S7. The asymmetric unit of **2**, thermal ellipsoids drawn at the 50% probability level

Crystal Data. $C_{18}H_{10}N_4$, M_r = 282.30, monoclinic, $P2_1/n$ (No. 14), a = 4.7982(2) Å, b = 9.7831(5) Å, c = 13.4869(9) Å, b = 97.129(5)°, a = g = 90°, V = 628.20(6) Å^3, T = 100(2) K, Z = 2, Z' = 0.5, m(Cu K_a) = 0.738, 4598 reflections measured, 1138 unique (R_{int} = 0.0207) which were used in all calculations. The final wR_2 was 0.1159 (all data) and R_1 was 0.0412 (I≥2 s(I)).

Table S1. Crystallographic data of compound **2**.

**Compound**	**GP080 / 2021ncs0612**
Formula	$C_{18}H_{10}N_4$
$D_{calc.}$/ g cm^{-3}	1.492
m/mm^{-1}	0.738
Formula Weight	282.30
Colour	dark orange
Shape	needle-shaped
Size/mm^3	0.26×0.06×0.02
T/K	100(2)
Crystal System	monoclinic
Space Group	$P2_1/n$
a/Å	4.7982(2)
b/Å	9.7831(5)
c/Å	13.4869(9)
a/°	90
b/°	97.129(5)
g/°	90
V/Å^3	628.20(6)
Z	2
Z'	0.5
Wavelength/Å	1.54178
Radiation type	Cu K_a
Q_{min}/°	5.601
Q_{max}/°	68.192
Measured Refl's.	4598
Indep't Refl's	1138
Refl's I≥2 *s*(I)	1005
R_{int}	0.0207
Parameters	100
Restraints	0
Largest Peak	0.237
Deepest Hole	-0.242
GooF	1.055
wR_2 (all data)	0.1159
wR_2	0.1120
R_1 (all data)	0.0459
R_1	0.0412

S5. Solid state luminescence studies

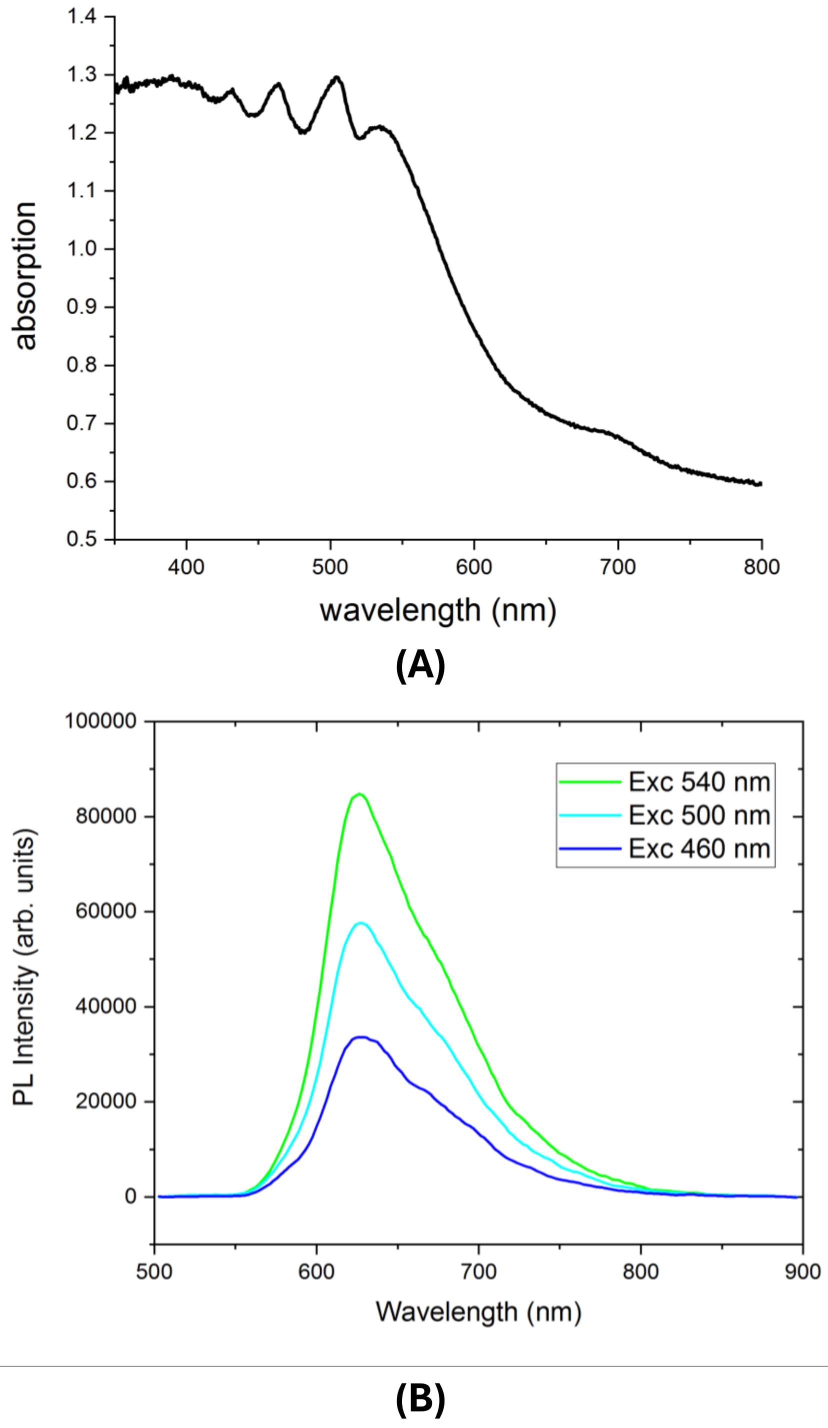

Figure S8. Absorption (A) and emission(B) spectrum of **2** in the solid state.

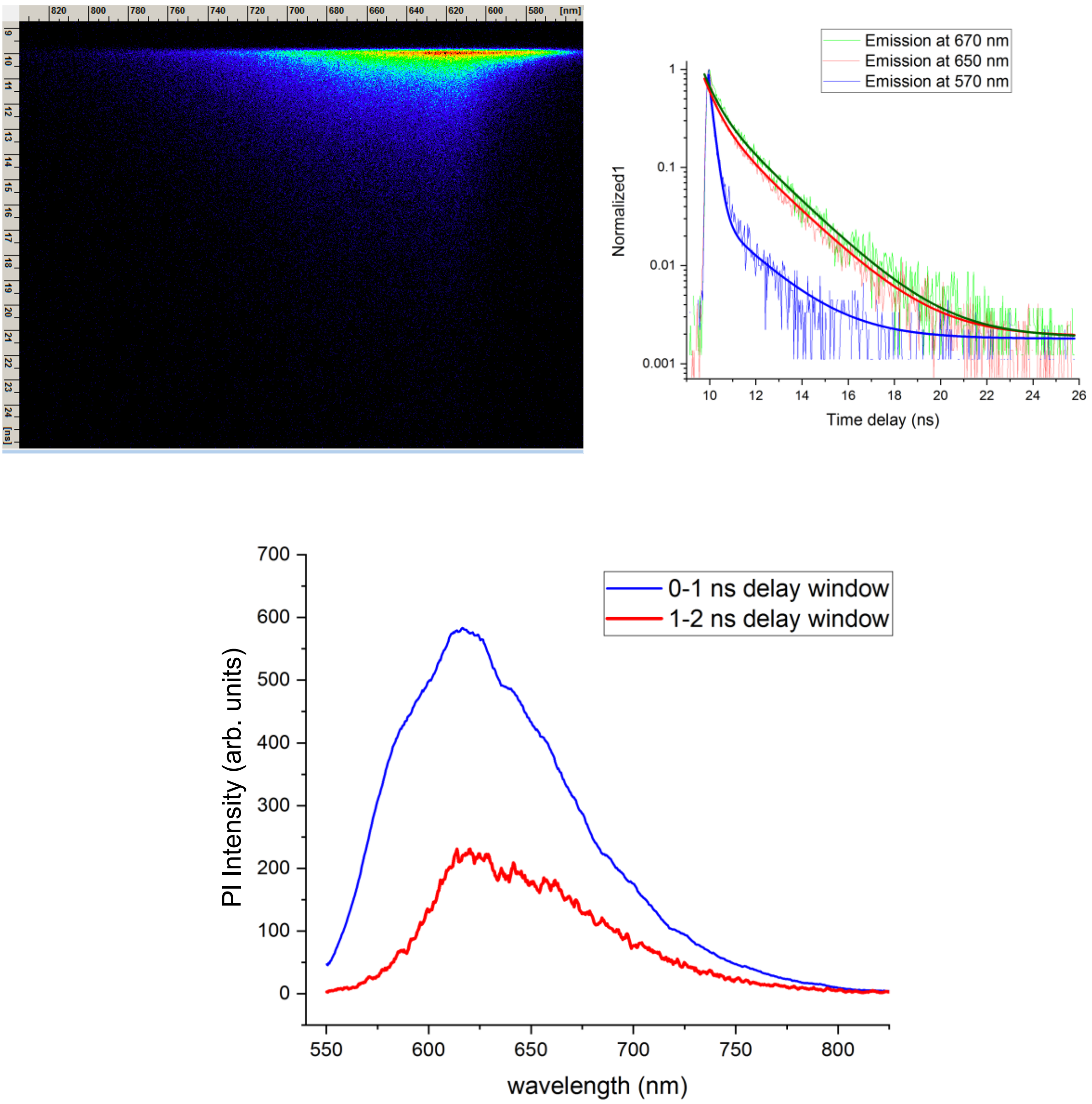

Figure S9. Time-resolved luminescence of **2** (λexc = 540 nm).

S6. Solution luminescence studies

Table S2. Relative quantum yields of **2** using Fluorescein in NaOH 0.1 M as standard (Φ = 0.95) in different solvents.

**Solvent**	**Φ**
Acetonitrile	0.90
Dimethylsulfoxide	0.85
Ethanol	0.96
Hexane	0.85
Toluene	0.92

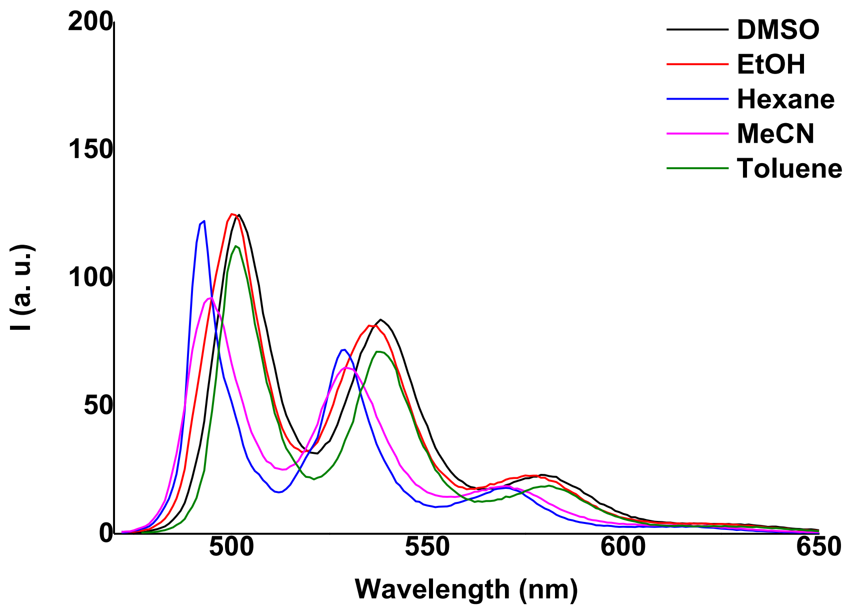

Figure S10. Emission spectra of compound **2** in different solvents.

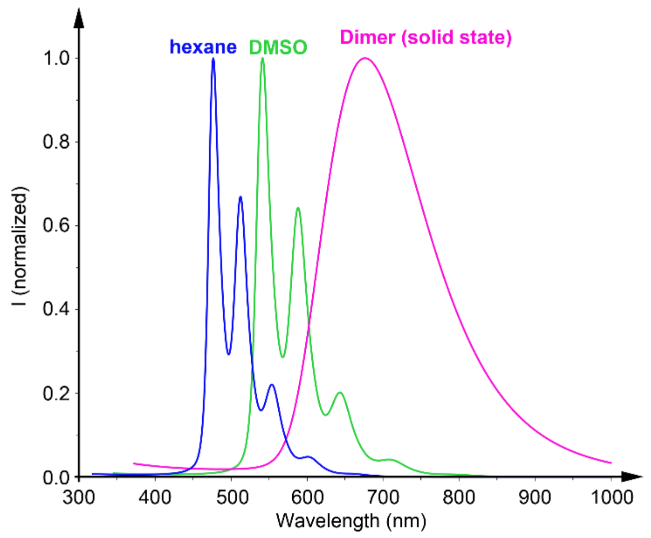

Figure S11. Theoretical emission spectra of **2** in various solvents and solid state at the B3LYP-D4/def2-TZVP level of theory. Normalized intensities were used.

S7. Theoretical calculations of the reaction mechanism

All geometry optimizations were performed without imposing symmetry constraints using the ORCA 5.0.3 software package [2]. The calculations employed the BP86 generalized gradient approximation (GGA) functional [3, 4] in conjunction with the def2-TZVP basis set [5]. To account for dispersion interactions, which are not adequately described by standard GGA functionals, the semi-classical D4 dispersion correction was included [6]. Solvent effects (dimethyl sulfoxide, DMSO) were incorporated via the Conductor-like Polarizable Continuum Model (CPCM) [7] throughout the geometry optimizations. The nature of each stationary point (minimum or transition state) was confirmed by analytical frequency calculations at the same level of theory, revealing no imaginary frequencies for minima and a single imaginary frequency for transition states. Time-Dependent Density Functional Theory (TD-DFT) calculations at the B3LYP/def2-TZVP level of theory [8,9] were performed on the optimized S_0 structures to simulate the emission spectra. The Cartesian coordinates for all optimized structures are provided in paragraph S8.

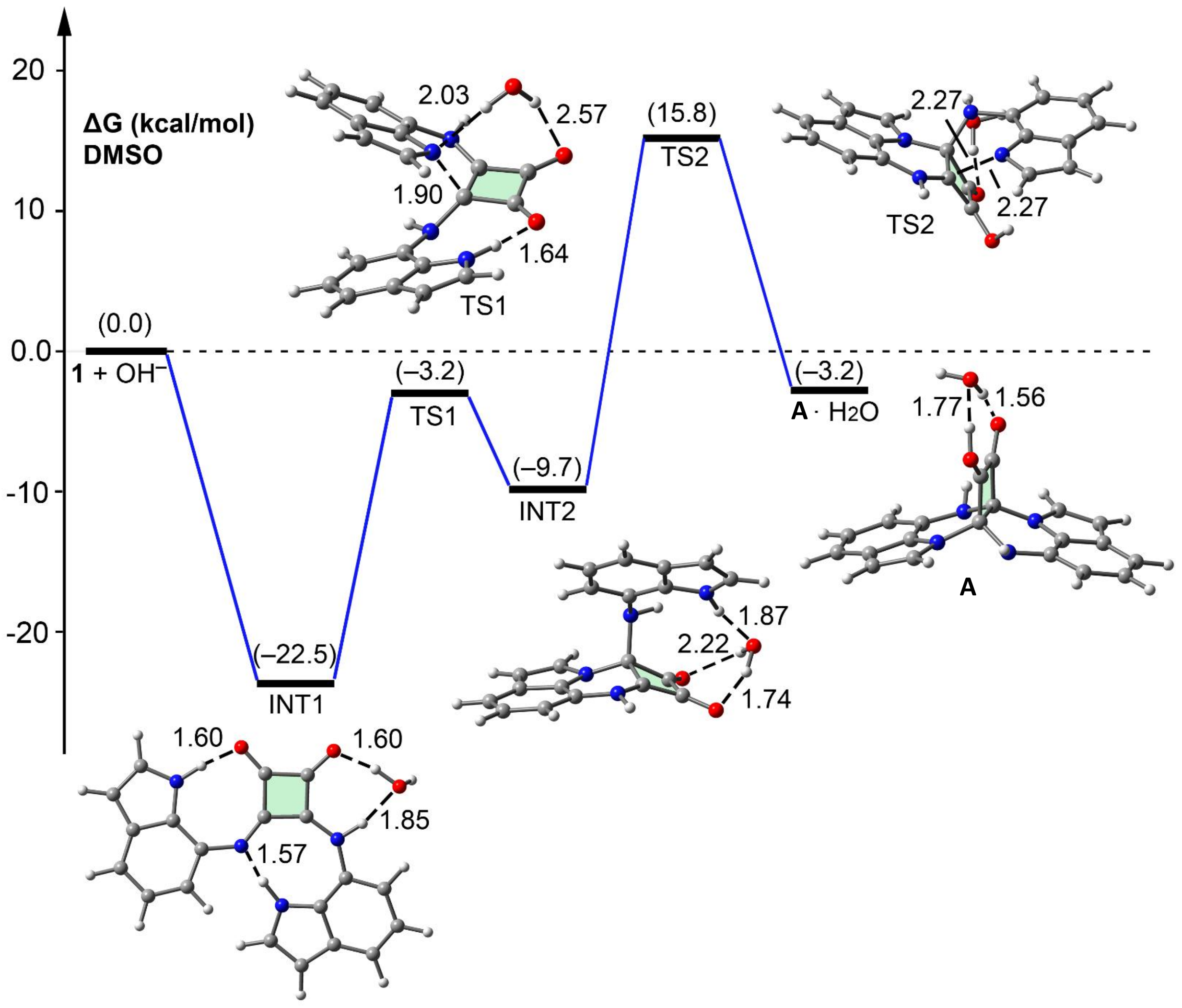

Figure S12. Energetic profile for the transformation of compound **1** into species **A** via two intermediates (INT1 and INT2) and transition states (TS1 and TS2). Distances in Å.

The energetic profile shown in Figure S12 represents a stoichiometric consistent model where the enol form includes hydroxyl leaving group that partially deprotonates the enol's hydroxyl group $[RO\cdots H\cdots OH]^{-}$, which is not detailed in Scheme 2 of the main text for simplicity. This approach allows for the direct computation of the ΔG values. In contrast, the energetic profile in Figure S12 is a simplified representation where the enol compound, without the presence of the hydroxyl group, is assigned a relative energy of zero. This allows for a more straightforward evaluation of the enol-keto energy difference and the thermodynamics of subsequent oxidation reactions.

Figure S13. presents the energy profile for the transformation of compound **A** (enol form) into **2**. The initial step involves conversion to the keto form, which is significantly more stable than the enol form, with an energy difference of –12.2 kcal/mol. Aerobic oxidation involving O_2 can be postulated, forming the diketo derivative B. This derivative is thermodynamically favored by –25.8 kcal/mol, considering the formation of H_2O_2 as a by-product of the oxidation. This analysis indicates that the diketo form, B, is more stable than INT2. The final step, which is not studied in detail here, involves photodecarbonylation induced by visible light, a process extensively studied in previous works. This step is highly exergonic, driven by both the aromatization of the system and entropic contributions.

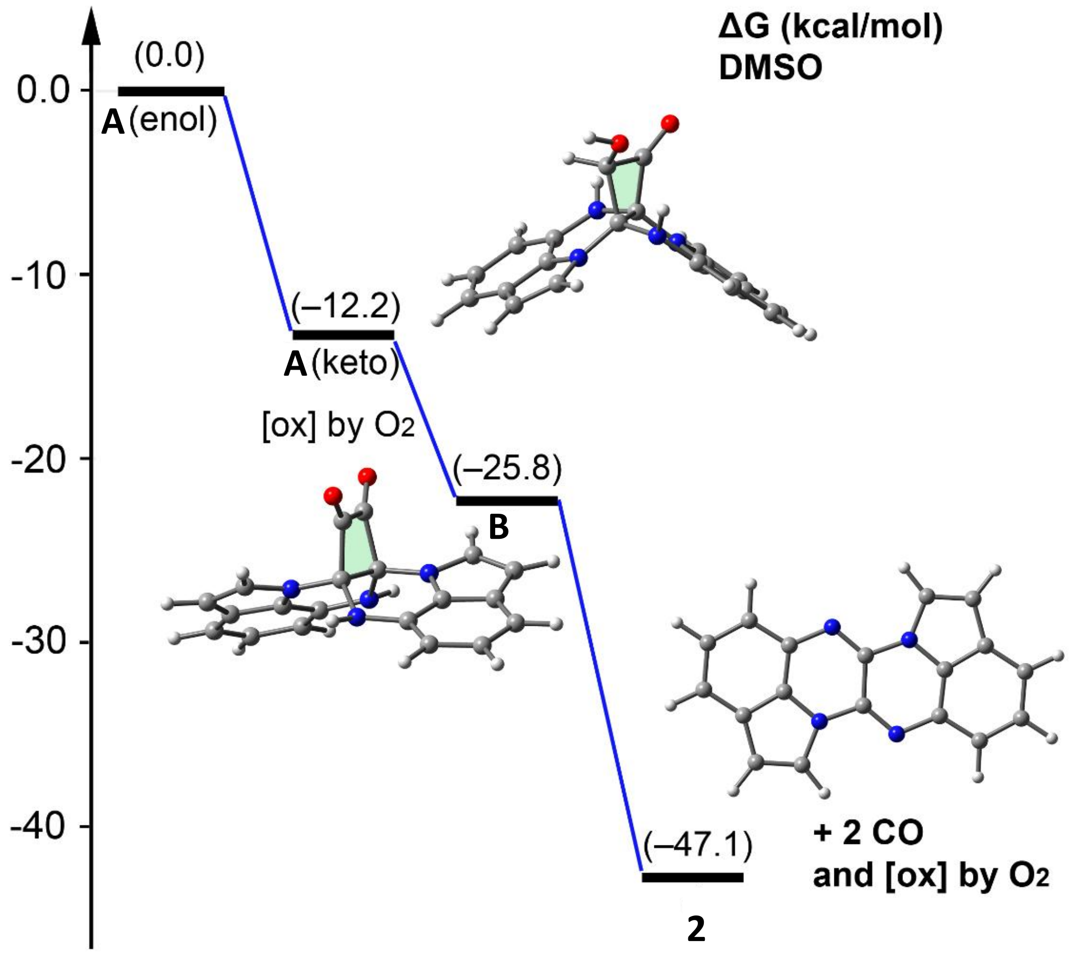

Figure S13. Energetic profile for the transformation of compound **A** into **2** via compound **B**.

[2] Neese, F. The ORCA program system. WIREs Comput Mol Sci. 2012, 2, 73-78. https://doi.org/10.1002/wcms.81

[3] Becke, A. D. Density-functional exchange-energy approximation with correct asymptotic behavior. Phys. Rev. A 1988, *38*, 3098–3100. https://doi.org/10.1103/PhysRevA.38.3098

[4] Perdew, J. P. Density-functional approximation for the correlation energy of the inhomogeneous electron gas. Phys. Rev. B 1986, *33*, 8822–8824. https://doi.org/10.1103/PhysRevB.33.8822

[5] Weigend, F.; Ahlrichs, R. Balanced basis sets of split valence, triple zeta valence and quadruple zeta valence quality for H to Rn: Design and assessment of accuracy. Phys. Chem. Chem. Phys. 2005, *7*, 3297–3305. https://doi.org/10.1039/B508541A

Barone, V.; Cossi, M. Quantum Calculation of Molecular Energies and Energy Gradients in Solution by a Conductor Solvent Model. J. Phys. Chem. A 1998, 102, 1995–2001. https://doi.org/10.1021/jp9716997

[7] Becke, A. D. Density-functional thermochemistry. III. The role of exact exchange. J. Chem. Phys. 1993, *98*, 5648–5652.

[8] Lee, C.; Yang, W.; Parr, R. G. Development of the Colle-Salvetti correlation-energy formula into a functional of the electron density. Phys. Rev. B 1988, 37, 785–789.

S8 Cartesian Coordinates and thermodynamic data

1, ΔH=-1140.25804462 Eh , TΔS=-0.06121479 Eh, NImag = 0

```
C   -5.73038568419242     1.50182675641490     1.54080184167481
C   -9.11639249069846     4.44971689942499     0.26048357256240
C   -8.20008375194366     4.46394085802087     1.32829083626654
C   -6.75003661473595     2.37072517304228     1.04072351232448
C   -10.10550436926244     5.48612596519374     0.14246089628205
C   -8.28933021149336     5.51870697842826     2.24668514055935
C   -9.25065674740705     6.53263387830505     2.12936045509177
C   -10.16504439923883     6.52558934880812     1.08346018860676
H   -7.58640471162665     5.54503860729457     3.08438973261830
H   -9.27223376095792     7.32797908485585     2.87448161439908
H   -10.91504886239039     7.31196611979694     0.99214595160105
N   -9.29460402686840     3.58700466105010    -0.79000010685376
N   -7.18151362969623     3.51333692855502     1.59840624947037
H   -6.64583570302719     3.80795214495488     2.41521002275931
C   -10.86968785392477     5.18557357447050    -1.02819609676649
C   -10.34430265342231     4.03117117316138    -1.56157791583405
H   -10.63548538889071     3.47546827650234    -2.44763813062146
H   -11.70331662883513     5.75580982421882    -1.42441381708557
C   -6.94854529608868     1.51027609073393    -0.15486677167521
C   -5.85517317587827     0.61275718290151     0.35632183771548
O   -7.67466528963516     1.48731791026052    -1.14620916454894
O   -5.32099727550490    -0.40762030855218    -0.07285887680232
N   -5.06384552390863     1.62315837018745     2.70006018209843
C   -3.99759175814661     0.94343647478785     3.34470763975312
C   -3.29441222228951    -0.17997014166885     2.87255481314507
C   -3.61502302639826     1.47520585313562     4.58356060788218
C   -2.21996627603117    -0.73923772027890     3.64676171775400
C   -2.57080050485615     0.92734064051746     5.34192322836188
H   -4.14920945166449     2.34992771196391     4.96509636282887
C   -1.86476514440744    -0.17765638989048     4.88306612192075
C   -1.72041464441376    -1.85240336942910     2.90098896674697
C   -2.47342390298969    -1.93287413838266     1.75206908575868
H   -2.31733553170099     1.38381621681503     6.29873382913215
H   -1.05040518044180    -0.60373874116352     5.46992640997204
H   -0.90595272083597    -2.51119469586976     3.18337844207103
N   -3.41648698442167    -0.93049193868884     1.73195437621437
H   -2.41733791559911    -2.63709382825480     0.92762645796572
```

H	-4.12679171770842	-0.79875112622016	0.97911866741053
H	-8.71310870898090	2.74811734884836	-1.00544946607293
H	-5.40533025948649	2.37286771574900	3.30189813531316

INT1, ΔH=-1216.26691788 Eh , TΔS= -0.06656233 Eh, NImag = 0

C	-5.93502798969639	0.90026985241130	1.26383624816103
C	-5.78359103092879	4.15741478184519	3.14372695899346
C	-7.08489596532636	3.98434829669255	2.62615628999678
C	-7.06748277435952	1.83050931850925	1.22122140466978
C	-5.44444878657827	5.30788716299849	3.93589776967099
C	-8.03332466691539	4.96619998138833	2.96170165550747
C	-7.70977679358525	6.08314102826979	3.74986164232226
C	-6.42130104561365	6.27423024331308	4.23760698258406
H	-9.04956188337281	4.84553394894586	2.58124065219074
H	-8.49251956974194	6.81059733125825	3.97414995194070
H	-6.17184572663591	7.15034743296775	4.83926941669389
N	-4.65835594436517	3.38751574812989	3.00461581468697
N	-7.54158831884924	2.97039415720674	1.76363742919442
H	-8.49264488052981	3.19121725159410	1.40286044679819
C	-4.05504516538197	5.17333700094176	4.25598497398831
C	-3.62658948348941	3.99290704145778	3.67883767968885
H	-2.64515358344092	3.52676096866563	3.69267018226862
H	-3.45043585146122	5.86366078419889	4.83718619192658
C	-7.77946251499123	1.02380850410009	0.23901280644586
C	-6.64457408327539	0.07031379785462	0.20847289015897
O	-8.89029286812052	1.10508627835434	-0.34003999449356
O	-6.39726922812800	-0.95584114827875	-0.44645181190430
N	-4.86608342151615	0.93627502775782	2.01473168065893
C	-3.77055972683277	0.08629101619175	2.14607827168494
C	-3.32043840037726	-0.96163561559626	1.29761778499519
C	-2.95030409211359	0.33879188112139	3.26634020733301
C	-2.09478929530596	-1.66819818022034	1.54367961587484
C	-1.75877827299582	-0.35718676463766	3.52400718163133
H	-3.28143511746327	1.11231132664908	3.96010942599872
C	-1.30811866612813	-1.35500446258104	2.66697958268754
C	-1.95637089704517	-2.62554737490088	0.48552109786263
C	-3.06136677839258	-2.47997712516384	-0.32831137049018
H	-1.17952082841407	-0.09871876271669	4.41319826727154
H	-0.37513173945330	-1.88782794579716	2.86001963670055
H	-1.14092179596107	-3.32990475491654	0.34636652666433
N	-3.86950656744482	-1.47571570342652	0.14709148220843
H	-3.34462900264161	-3.02424088620585	-1.22479229198909
H	-4.85415422320421	-1.29390658428994	-0.17705579107563
H	-4.66678502375837	2.37204178197572	2.61485578883852
O	-10.12730514976501	3.23294690096837	0.53319664803638
H	-9.75828004138692	2.36746493129015	0.13276547999274
H	-10.08916981501277	3.87122004167351	-0.19788759637478

TS1, ΔH=-1216.22550951 Eh , TΔS= -0.07058763 Eh, *f*=-273.1, NImag = 1

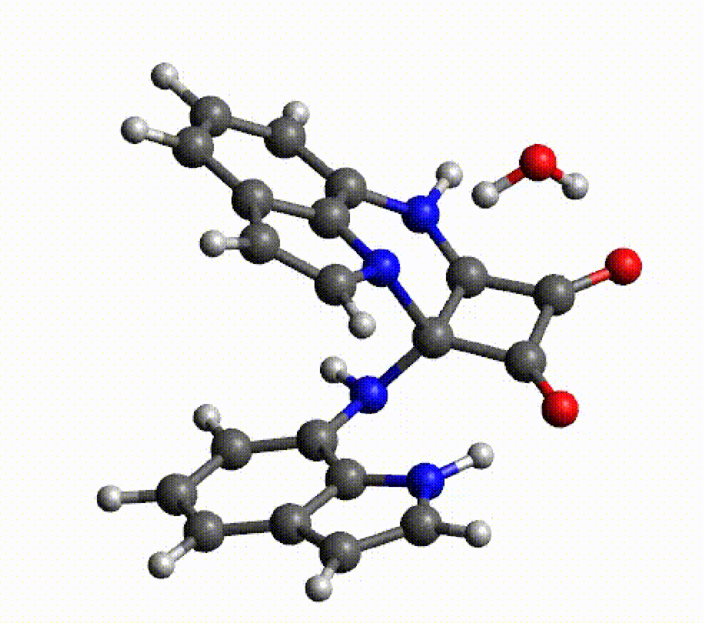

C	-0.91081753692292	0.03399791570613	-1.55054840103336
C	-0.69090184350381	1.80904780601400	0.34478695624140

C	-2.06875387286125	2.05627669111846	0.20733656750161
C	-2.02260106392987	0.90543112635176	-1.88907181782237
C	0.00273582235814	2.04936052912198	1.56786569519666
C	-2.74757432305304	2.61613034199162	1.29546541574484
C	-2.05425690919172	2.91729741874105	2.48486246186352
C	-0.69491877029193	2.63781349039884	2.63994266011480
H	-3.81903484143999	2.81405985881000	1.21855436965168
H	-2.60631509970419	3.37209524215279	3.31020144445503
H	-0.18695220392613	2.87921791237614	3.57585368135001
N	0.16740480127947	1.29407351339177	-0.61357700649741
N	-2.69869269940000	1.69306743527264	-1.00130904663437
H	-3.46585755585280	2.26239947485801	-1.34713841284279
C	1.35053416552818	1.61530978543434	1.32465558963465
C	1.38951776836841	1.14106853052088	0.02532805919668
H	2.22882889078328	0.71163670992603	-0.51490526524506
H	2.18570967535971	1.65257786218285	2.01919374530465
C	-1.83039792080454	0.84367136015678	-3.31821920022470
C	-0.65735723676982	-0.07976593340474	-3.05053399712258
O	-2.34949463480583	1.36214232343098	-4.31792730120013
O	0.24101687277730	-0.59420760658035	-3.72795274695378
N	-1.04266157940097	-1.00845621796626	-0.60898397951192
C	0.01178348213895	-1.65741929680218	0.05871029451285
C	1.24272719439184	-1.99800622683115	-0.53779814607156
C	-0.16028733671233	-2.01803622567816	1.39936012032429
C	2.27990883631993	-2.64589892212016	0.20777263976120
C	0.84283647490055	-2.67578315316895	2.13236673144339
H	-1.09135718868471	-1.73383070395980	1.89537883754936
C	2.06844065234759	-2.98812885970873	1.55716664874659
C	3.38866589106609	-2.79301191436397	-0.68822597548282
C	2.99954650199217	-2.25311301202524	-1.89808144944008
H	0.65385702894405	-2.91790441995042	3.17973310803204
H	2.85228906095553	-3.48017525185601	2.13603612332154
H	4.35181351417828	-3.24218256586386	-0.46601387030287
N	1.71322930479762	-1.77791195980781	-1.81239492388446
H	3.54585362385239	-2.16872484843469	-2.83314292662994
H	1.18408352552090	-1.33877467955196	-2.60756432804644
H	-1.80091195773641	-0.80897224620936	0.04282191577263
O	0.19249871677611	3.29217289729149	-2.87888860662920
H	-0.44005828144215	2.87554448284025	-3.49202489301005
H	0.23263905179793	2.66449973619498	-2.12068597113356

INT2, ΔH=-1216.23681953 Eh , TΔS= -0.06898397 Eh, NImag = 0

C	-5.87931960424491	0.70426603495631	0.58407995871441
C	-3.72541299417708	1.42518199709231	-0.26996660600901
C	-3.92439174294665	2.77535365116577	0.06614752373941
C	-6.17025659279326	2.17374256152617	0.48416520269945
C	-2.50205041441362	0.89066357082656	-0.73649137325166
C	-2.82813838303037	3.62529491017314	-0.09326813150491
C	-1.60181556970649	3.12035667085086	-0.58517297085661
C	-1.41620598454336	1.77988957770007	-0.91293232207599
H	-2.92206194588603	4.68367085920042	0.15722833493847
H	-0.77193131888352	3.81903947176451	-0.71289003333566
H	-0.45616221244727	1.42867465061782	-1.29527017594720
N	-4.66100062982486	0.41418648896168	-0.17531475914823
N	-5.19508943310610	3.14222257344811	0.51464163206489
H	-5.46458768824416	4.12063278641110	0.50047593293815
C	-2.74050142353575	-0.51652647683050	-0.90549954339246
C	-4.05073491810066	-0.76606576390749	-0.53264449424169
H	-4.60088379746885	-1.69973463098368	-0.47995965057883
H	-2.03627926538350	-1.26006316401408	-1.26834617494911
C	-7.55519175559030	2.05366306269224	0.21653962006535
C	-7.33844750356057	0.58204791694250	0.05529334990429
O	-8.57337723400043	2.80422133176217	0.17330259666149
O	-8.00054977218672	-0.39864803994489	-0.29612056330991
N	-5.78738322606412	0.04228649043546	1.92192381182427
C	-5.40871077203048	0.90715268470300	2.98248488211147
C	-6.36371692120732	1.58011367287203	3.76848030625635
C	-4.06348264133944	1.16781757591373	3.24813267925238
C	-5.98692011600584	2.45989373918401	4.83014487792567
C	-3.67789197700171	2.06072121924748	4.26622623951760
H	-3.31087330256333	0.66323842947449	2.64141914933188
C	-4.61965957008759	2.69575518044268	5.06985516968690
C	-7.20613341257202	2.96050074715365	5.39518844493641
C	-8.23912890129542	2.39950905988447	4.67425803826850
H	-2.61393820877286	2.24286049663445	4.43167128046848
H	-4.30613825778381	3.37713094981974	5.86385784031150
H	-7.30907838297871	3.65448376067454	6.22463286904422

N	-7.74412282368575	1.55759480422296	3.70478973074990
H	-9.31231055027437	2.54422836409120	4.75867524119019
H	-6.70039747627697	-0.37540134874039	2.11242821015237
H	-8.35161864409348	1.19039986570250	2.94500234591967
O	-9.77569608674445	0.95895637138832	1.75356140244711
H	-9.53777005631096	1.74567661997658	1.16824625165371
H	-9.57013795883571	0.21436865650763	1.15218876582649

TS2, ΔH=-1216.19604006 Eh , TΔS= -0.06967679 Eh, *f*=-113.0, NIMag = 1

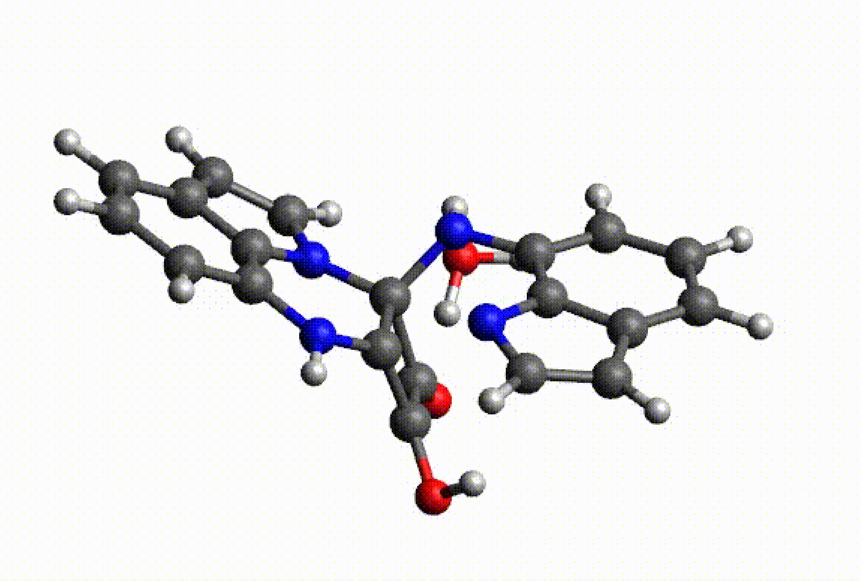

C	-0.06010574335295	-0.48594925370727	-0.57301010648654
C	1.23144329774234	0.65074706645160	-2.29177798336659
C	0.71542784120829	1.91977675701159	-2.00009717910912
C	-0.82475985896871	0.82627412241156	-0.55052466836487
C	2.26113844602554	0.39679656994068	-3.22505277791723
C	1.27946919783491	2.99621585092522	-2.68638232095719
C	2.30757192743054	2.77148942983656	-3.63267565520718
C	2.80333168813087	1.50261820778913	-3.92010665001900
H	0.91681272230096	4.00946774109913	-2.50322671223616
H	2.71928729540244	3.63541446252147	-4.15791188796763
H	3.58972295273466	1.36948118413897	-4.66523882735818
N	0.81367010886526	-0.53560415887650	-1.73551777753660
N	-0.35149736243230	1.98508409181767	-1.10588600023429
H	-0.84744387979504	2.85672244777723	-0.96238810573287
C	2.46203494764818	-1.02562648524718	-3.19061913378487
C	1.57987919200565	-1.55528674407846	-2.26436487116988
H	1.44021957652798	-2.57526757874656	-1.91530175762912
H	3.16891082354610	-1.60145694347483	-3.78103291002946
C	-2.06626878957308	0.17937444765340	-0.45026929457967
C	-1.51040042273192	-1.12314997804917	-0.61997468553687
O	-3.29408593846549	0.68218660104398	-0.15046998521240
O	-1.89191269438877	-2.29422060149596	-0.77834749590241
N	0.74350283187436	-0.83169877366815	0.61424052397178
C	0.09088803283226	-0.81957662480260	1.87058982107701
C	-0.63228363772816	0.31262323702995	2.30024641030586
C	0.16228649284815	-1.90840986115128	2.74584783180029
C	-1.37858541476090	0.29947978132952	3.52842691711119
C	-0.49266839463268	-1.88947000873587	3.99766690624366
H	0.74374945539827	-2.78672810433280	2.45318360056384
C	-1.27882090156319	-0.81182592233890	4.39292457187711
C	-2.07844228570216	1.54399057084927	3.54600129242934
C	-1.70573279623918	2.20602754893364	2.37047984384656
H	-0.39725195728845	-2.76013361459212	4.65156528941348
H	-1.81073558324371	-0.83437586171411	5.34719078962668
H	-2.73722573391753	1.92050614024158	4.32605854038165
N	-0.80286921034510	1.48774826366599	1.62183192049083
H	-1.99892323476935	3.21087388763633	2.05946355455916
H	1.12546477726724	-1.76748345176012	0.44119711560388
H	-3.15270952779520	1.22934882836644	0.66041827962752
O	0.24430259772930	-3.87505598100663	-0.30934591268867
H	-0.57721025569158	-3.36032501715969	-0.57410346270161
H	0.06099018803212	-4.09612251353264	0.61895451279857

A, ΔH=-1216.22944306 Eh , TΔS= -0.06660417 Eh, NImag = 1

C	-4.25270933426108	1.24700086488937	0.98522054513986
C	-3.23023727006492	1.45937375535547	-1.26393561451948
C	-3.23437047389595	2.85564661856658	-1.29090737902705
C	-4.25384527918500	2.83891671682481	0.97243974402692
C	-2.88029025770605	0.62872190612290	-2.35706800829723
C	-2.88487914630736	3.44791238996970	-2.51074588749729
C	-2.52364777372012	2.64699584982961	-3.61904116833480
C	-2.50456332146676	1.25517449143322	-3.56804974359205
H	-2.87636875937676	4.53680955907107	-2.60015842320337
H	-2.25179294969773	3.14963494053797	-4.55015096532542
H	-2.22243493403357	0.66911278698742	-4.44398854748248
N	-3.57030772642643	0.68038771959327	-0.18444996564911
N	-3.51672254752576	3.53918711444322	-0.11195853800718
H	-3.90618022151651	4.46727088029894	-0.27491911591646
C	-3.04356103415096	-0.71734000700099	-1.88109477727451
C	-3.47979032590045	-0.64250570825816	-0.56854691473981
H	-3.73715806960813	-1.43527557187036	0.12486191391294
H	-2.86405035449215	-1.63456609386659	-2.43398126760371
C	-5.73792525669581	2.71923123051592	0.86632437889781
C	-5.78693345089140	1.34370976279308	0.87948111454355
O	-6.64444867554022	3.74158930008522	0.84179850170329
O	-6.65392885802770	0.39726640098418	0.88888703896240
N	-3.76345906864133	0.52962330012784	2.17503067929160
C	-3.58391439884288	1.22127015199102	3.37152270606319
C	-3.53352490336661	2.61709602862416	3.34533766820756
C	-3.40610382472751	0.62352381940865	4.62523018503745
C	-3.30406902740576	3.43966340345910	4.47750759798699
C	-3.16480309892607	1.41555922620348	5.77113536926825
H	-3.44047828327350	-0.46482527346504	4.71243991948292
C	-3.10137779834531	2.80625439665645	5.72532377868553
C	-3.38480338721987	4.78924983316521	3.99182090362830
C	-3.65948726844663	4.72350335771136	2.63514320135063
H	-3.02450869128506	0.90680135482731	6.72769602861716
H	-2.91652362037724	3.38655494801785	6.63047368446186
H	-3.25657959833192	5.70211460257247	4.56599310440691
N	-3.73101980842208	3.40452321835021	2.23838377009934
H	-3.81334190559310	5.52604860044937	1.92154586907230
H	-4.30815197218936	-0.32607298070181	2.29619831133141
H	-7.53173194540424	3.28006282301595	0.94575496469264
O	-8.69034120840607	1.96793941190397	1.20655678612138
H	-8.85606686805225	1.94517032087215	2.16341943405359
H	-7.96387478225236	1.23370201950442	1.06655522742417

A(keto), ΔH=-1140.22619007 Eh , TΔS= -0.06527708 Eh, NImag = 1

C	-4.61376284604190	1.27896510655751	1.07870986565170
C	-3.26672719867686	1.59114755848154	-0.93864577495792
C	-3.66261473720905	2.91108553449644	-1.15147086214275
C	-4.66352885466221	2.88213436673510	1.11186206292270
C	-2.48418740412087	0.82776153742120	-1.83312841650967
C	-3.24455433697755	3.50337824951438	-2.34381471212891
C	-2.45118173256066	2.76966286049205	-3.25346997558470
C	-2.05618391900272	1.45314088315881	-3.02137285803288
H	-3.51869579466776	4.53539750685656	-2.56739297864349
H	-2.13505993903097	3.26364894155451	-4.17316541740336
H	-1.44675636799587	0.92129453684194	-3.75243855634025
N	-3.62006710327940	0.81630625066192	0.14001844543023
N	-4.44810925712549	3.51164783868786	-0.16415175620352
H	-5.06402756536507	4.27649523884124	-0.42023776832497
C	-2.38360585376045	-0.47736171677627	-1.23302383062173
C	-3.09428690307773	-0.45335020499560	-0.05104376188825
H	-3.24658541337860	-1.22120978038199	0.69986966602557
H	-1.83548566481842	-1.33192236531925	-1.61685821433404
C	-6.20911853840507	2.70129550702028	1.26924666475131
C	-6.13900665901972	1.27738709530853	0.66127062424143
O	-7.07939489968719	3.35543911618243	1.78156159160564
O	-6.97870023277692	0.31078228441194	1.24108744719689
N	-4.49649901926698	0.65082673778455	2.35781518637486
C	-3.75748032113967	1.27685124364288	3.37979068928793
C	-3.44423546358330	2.62220405146693	3.21639450723710
C	-3.32029882580759	0.67300534435694	4.55748734402085
C	-2.72441858680956	3.40633032572348	4.14414137739526
C	-2.59789188533745	1.42729002230655	5.50854226291866
H	-3.53420444950562	-0.38076270403786	4.74146320814285
C	-2.28435702421545	2.77325603722371	5.32291960663941
C	-2.69469397945924	4.73300550010304	3.58480456686295
C	-3.38078847518816	4.70068198317067	2.39043876166818
H	-2.27010769537301	0.92790712441799	6.42130758770981

H	-1.72630561767340	3.32073661665306	6.08307585081741
H	-2.22043979386149	5.61163824363601	4.00991270640677
N	-3.83234577901625	3.40834880620057	2.15614398018095
H	-3.56890743989058	5.48733191777596	1.66801806734340
H	-5.31889437194133	0.11246119762489	2.62889772201832
H	-7.18229228160130	-0.37136246374082	0.57888816012746
H	-6.22677955868883	1.31641809994018	-0.43726549986125

B, ΔH=-1139.02953529 Eh , TΔS= -0. 06516771 Eh, NImag = 1

C	-4.15954186324427	1.30355375352151	1.16472474848614
C	-3.34106115132887	1.46267578073254	-1.16242050150955
C	-3.01412793267697	2.81413127010038	-1.10536544555714
C	-4.17877509856162	2.87457394736202	1.03416191881875
C	-3.18831247616129	0.63472725254108	-2.29135337221589
C	-2.45575797661636	3.36255578884252	-2.26331864641470
C	-2.26121807067531	2.55376686490327	-3.40247485360861
C	-2.61900083612023	1.20580071637633	-3.44599067100412
H	-2.17360680119385	4.41606018353196	-2.28896335799020
H	-1.82168766487116	3.01344946625208	-4.28864255111614
H	-2.46757799836393	0.61978969592533	-4.35220244155917
N	-3.93333491670781	0.74484344859845	-0.14553375885732
N	-3.25755911982090	3.47529015095066	0.09910525203659
H	-3.32649024977445	4.48934564086137	0.04178668738402
C	-3.72731899976827	-0.64605214287571	-1.90636211206563
C	-4.17840307227263	-0.54520213185735	-0.61012132069407
H	-4.67029601562320	-1.27658002401649	0.02283274107425
H	-3.79130613759885	-1.53811274494175	-2.52153992996946
C	-5.73559031770311	2.78866750242350	0.77009235787376
C	-5.73143116803407	1.34275739374603	1.33227631193435
O	-6.52616184153574	3.55389909878451	0.27806935175948
O	-6.52788508973007	0.55397819979263	1.77522781923529
N	-3.27989358890262	0.72897738855170	2.15443296407352
C	-3.12807090188882	1.39672299002630	3.37039186687104
C	-3.49666130170950	2.73814705761218	3.40618672391089
C	-2.62396611300890	0.86411603989552	4.56004721809319
C	-3.43489068013484	3.56990620489309	4.54096633217629
C	-2.52078388889409	1.67775764535305	5.70767110274250
H	-2.31352153396397	-0.18075831148900	4.60359965107962
C	-2.91901888507063	3.01492042271381	5.72817515053068
C	-3.98713368028324	4.83459502874936	4.12275024129830
C	-4.35769656392031	4.72084026390796	2.80226693241521
H	-2.12157279672947	1.23061244814770	6.61887933150795
H	-2.83845216828620	3.60448084233197	6.64115004406530
H	-4.11309705726785	5.72438442442794	4.73155156829137
N	-4.04884298084127	3.43848904126426	2.35503469138671
H	-4.83155861452321	5.43806953753715	2.14000000180040
H	-3.32442354619204	-0.28655063547795	2.20976005371638

2, ΔH=-911.14221116 Eh , TΔS= -0.05521726, NImag = 1

C	-3.27356497161912	1.36080906113250	1.18114842524667
C	-3.20340867553448	1.49127786266443	-1.21374126721037
C	-3.22266587734464	2.88274719398721	-1.22467737051322
C	-3.29085536878756	2.85029875183421	1.06464439910704
C	-3.15815184395606	0.64368689196619	-2.32958725840145
C	-3.19363442769284	3.47514590774712	-2.49664648866961
C	-3.14763151762705	2.66445244102448	-3.64730959332751
C	-3.12932030595582	1.26515025891744	-3.59323043268506
H	-3.20723807592269	4.56243463740886	-2.57949168340892
H	-3.12560423842176	3.15128397225221	-4.62328930951136
H	-3.09321702109043	0.67993412131327	-4.51223951312601
N	-3.22754188391136	0.73801368515610	-0.05899009230166
N	-3.26833521718597	3.57387118100129	-0.02251785134699
C	-3.15572689521870	-0.69922310726148	-1.78982144911459
C	-3.19805113838636	-0.61138086792236	-0.41820477741751
H	-3.21104537725340	-1.37427770246603	0.35109877209847
H	-3.12553640808693	-1.62746924488203	-2.35208420815127
N	-3.29576566771359	0.63724422182540	2.26831923455272
C	-3.34169902937057	1.32835804853824	3.47047197878352
C	-3.36101354247941	2.71983072857389	3.45953849688655
C	-3.37099923004103	0.73596150341585	4.74243928966549
C	-3.40607538536929	3.56742171876302	4.57538860483439
C	-3.41710575166230	1.54665208317560	5.89309865815075
H	-3.35754942208820	-0.35133097001021	4.82527149176317
C	-3.43521346279196	2.94595783021403	5.83902326532161
C	-3.40879671058731	4.91033142366444	4.03561702543396
C	-3.36600765609954	4.82249353294276	2.66401481056585
H	-3.43954416694595	1.05981633694238	6.86906628718896

H	-3.47109611110253	3.53118637131497	6.75803284298466
H	-3.43857303918427	5.83858248456964	4.59789302450487
N	-3.33676269373659	3.47309113592975	2.30479285050641
H	-3.35339848683219	5.58538570626679	1.89470023759040

S9 LC-MS studies of the reaction mechanism

Squaramide **1** was dissolved in DMSO to a concentration of 5 mM (2 mL total volume) and 10 equivalents of base were added (TBA-F, TBA-Cl, TBA-Br, TBA-I, TBA-NO_3, TBA-H_2PO_4, TBA_2SO_4, triethylamine, DBU, or TBAOH (TBAOH was added from a 1 M stock in MeOH)). In general, the vials were left open to air and placed on a stirring hotplate under the light provided by the fume hood, and were heated to 80 °C using a sand bath for 2.5 hours whilst stirring. For room temperature conditions (25 °C), the vial was not heated but all other conditions remained the same. For inert conditions, the vial was sealed with a rubber septum and placed under an argon atmosphere using a balloon. For dark conditions, the vial was covered in aluminium foil and placed in a dark fume hood. Each reaction was repeated 3 times.

To estimate conversion after 2.5 hours, 100 µL of the reaction mixture was diluted into 1 mL mass spec grade MeCN, and the solution was immediately subjected to LC-MS (10 µL injection). LC-MS traces were collected on a Thermo Fisher Scientific Vanquish Flex UHPLC with variable wavelength detector and ISQ EC mass spectrometer, using a Hypersil GOLD C18 column (150 mm length, 3.0 mm diameter, 3 µm particle size). 'Solvent A' was 0.1% (v/v) HCOOH in water and 'solvent B' was 0.1% (v/v) HCOOH in acetonitrile. Gradient was from 10% B to 90% B in 15 min, followed by 4 minutes at 90% B. The detection wavelength was set at 250 nm (UV-Vis1) and 450 nm (UV-Vis2). Squaramide **1** and its degradation products are visible at 250 nm, whereas product **2** is more prominent at 450 nm. The area under the curve for the peak at 450 nm corresponding to product **2** was used to estimate conversion – using the area under the curve for a sample of pure **2** dissolved in 10% DMSO in MeCN at 0.45 mM as a reference for 100% conversion (Figure S15). The identity of the peaks shown on the HPLC traces was confirmed by the mass spectrometry data. When **1** was degraded, but not converted to **2**, the degradation products eluted at the same time as the TBA+ ion and the masses of the degradation products could therefore not be accurately determined. All obtained HPLC traces are shown in Figures S16-S31, along with the observed mass for each peak. When conversion was checked after 5 h or 24 h, conversion appeared to decrease because compound **2** precipitated out of solution (in addition, the dark and inert conditions do form compound **2** over very long periods of time). When conversion was checked at shorter time, the reaction was not complete. We therefore decided to check the reaction after 2.5 hours.

For the photographs, 100 µL (from 0.45 mM HPLC-MS sample) was dissolved in 1 mL MeCN and a picture was taken upon irradiating the vial with a standard laboratory UV lamp (365 nm).

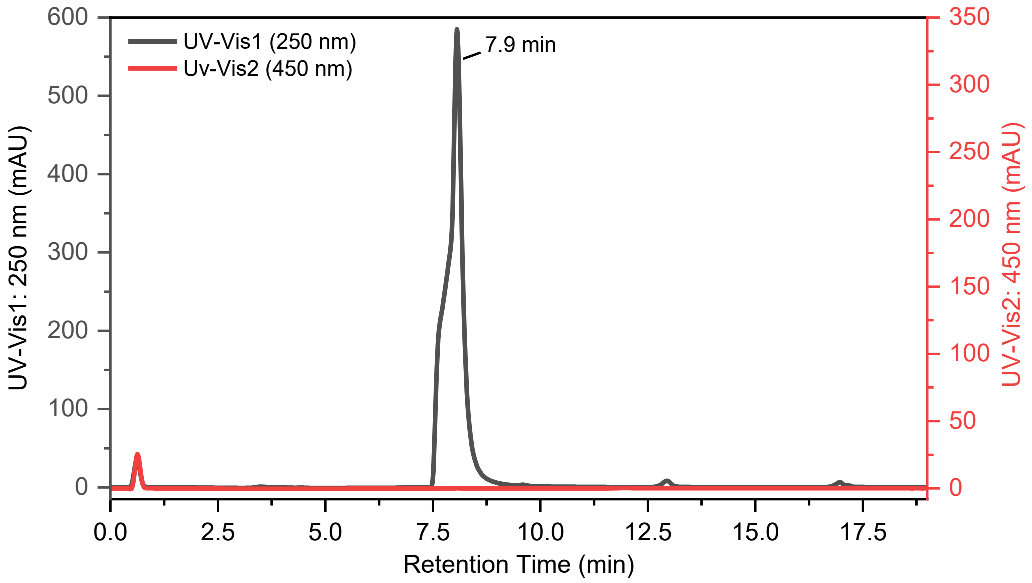

Peak at 7.9 min:
m/z (ESI+) = 343.1 [**1**+H]+
m/z (ESI-) = 341.1 [**1**-H]-
AUC = 221.06

Figure S14. HPLC trace of squaramide **1** showing the absorbance at 250 nm (black traces) and 450 nm (red traces). On the right is shown the dominant *m/z* value for the peaks observed in the HPLC traces, as well as their 'area under the curve' (AUC) in mAU*min.

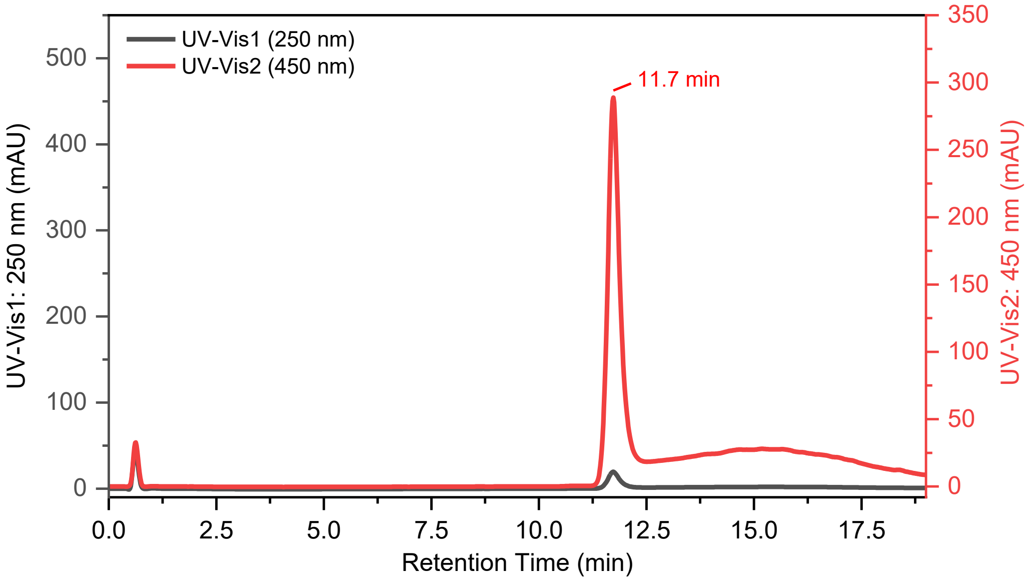

Peak at 11.7 min:
m/z (ESI+) = 283.1 [**2**+H]+
AUC = 102.26

Figure S15. HPLC trace of product **2** showing the absorbance at 250 nm (black traces) and 450 nm (red traces). On the right is shown the dominant *m/z* value for the peaks observed in the HPLC traces, as well as their 'area under the curve' (AUC) in mAU*min.

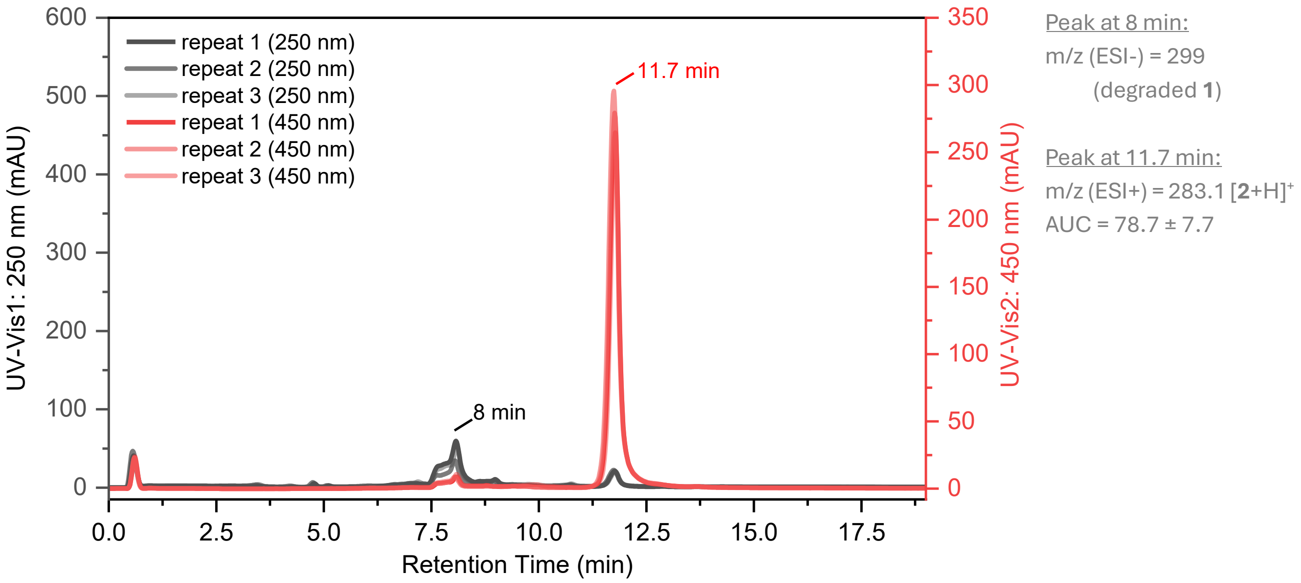

Figure S16. HPLC trace of the reaction mixture of **1** (5 mM) in DMSO in the presence of 10 equivalents TBAF after 2.5 h at 80°C under open air and light, showing the absorbance at 250 nm (black traces) and 450 nm (red traces). On the right is shown the dominant *m/z* value for the peaks observed in the HPLC traces, as well as their 'area under the curve' (AUC) in mAU*min if appropriate.

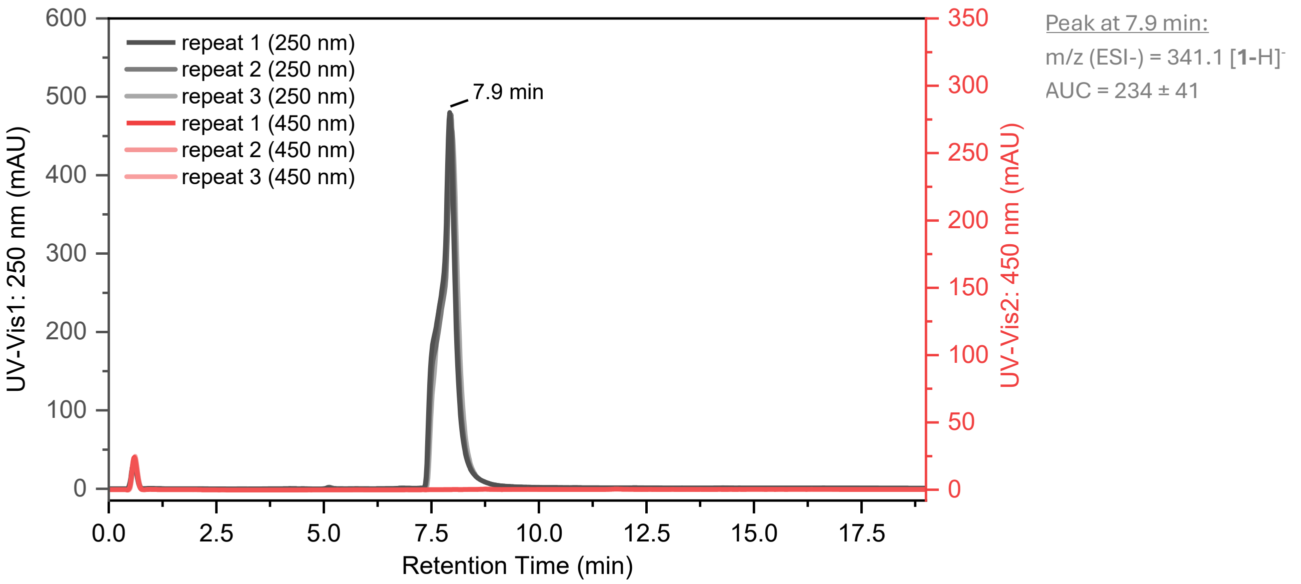

Figure S17. HPLC trace of the reaction mixture of **1** (5 mM) in DMSO in the presence of 10 equivalents TBACl after 2.5 h at 80°C under open air and light, showing the absorbance at 250 nm (black traces) and 450 nm (red traces). On the right is shown the dominant *m/z* value for the peaks observed in the HPLC traces, as well as their 'area under the curve' (AUC) in mAU*min if appropriate.

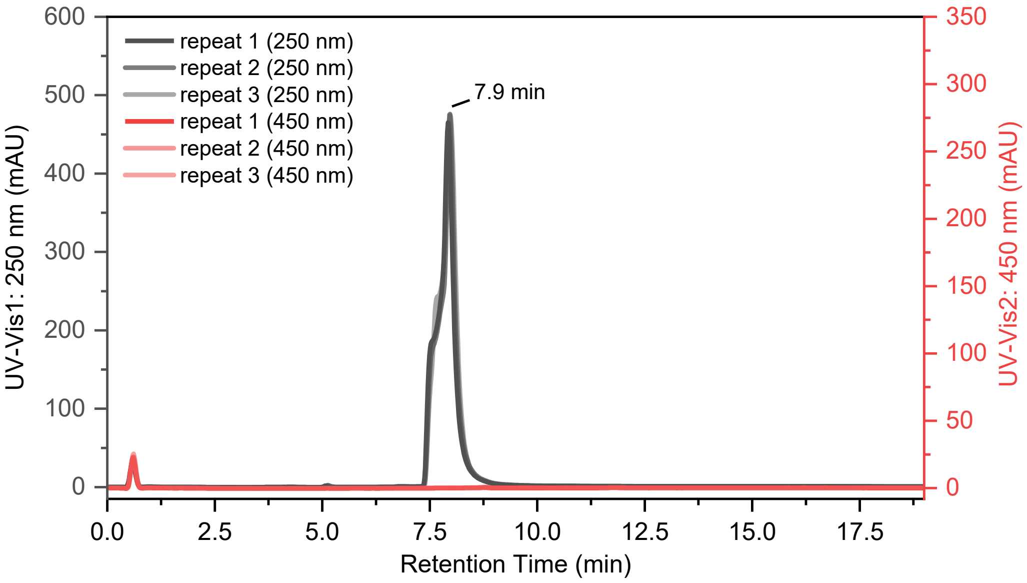

Peak at 7.9 min:
m/z (ESI-) = 341.1 [**1**-H]$^-$
AUC = 212 ± 10

Figure S18. HPLC trace of the reaction mixture of **1** (5 mM) in DMSO in the presence of 10 equivalents TBABr after 2.5 h at 80°C under open air and light, showing the absorbance at 250 nm (black traces) and 450 nm (red traces). On the right is shown the dominant *m/z* value for the peaks observed in the HPLC traces, as well as their 'area under the curve' (AUC) in mAU*min if appropriate.

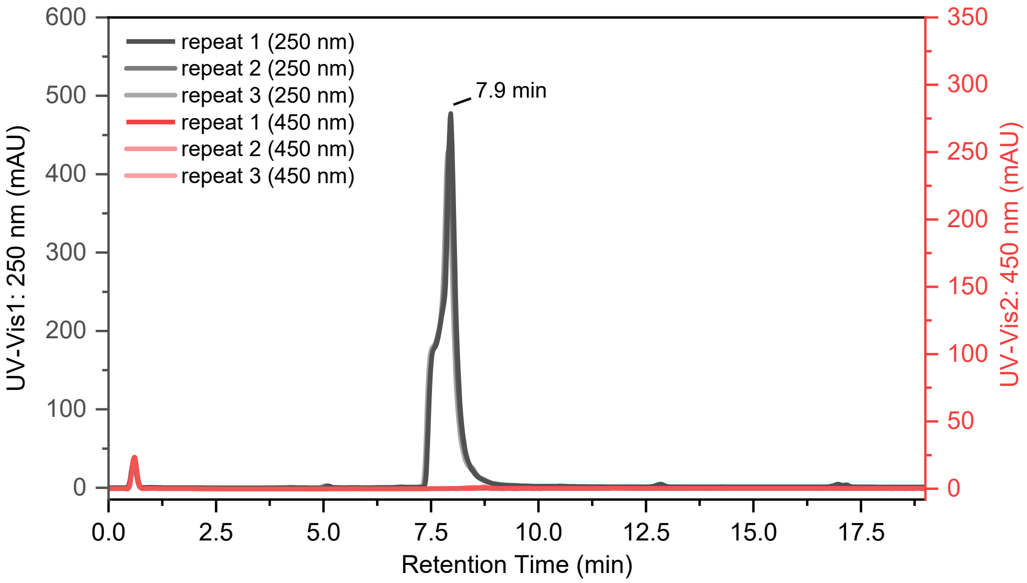

Peak at 7.9 min:
m/z (ESI-) = 341.1 [**1**-H]$^-$
AUC = 199 ± 5

Figure S19. HPLC trace of the reaction mixture of **1** (5 mM) in DMSO in the presence of 10 equivalents TBAI after 2.5 h at 80°C under open air and light, showing the absorbance at 250 nm (black traces) and 450 nm (red traces). On the right is shown the dominant *m/z* value for the peaks observed in the HPLC traces, as well as their 'area under the curve' (AUC) in mAU*min if appropriate.

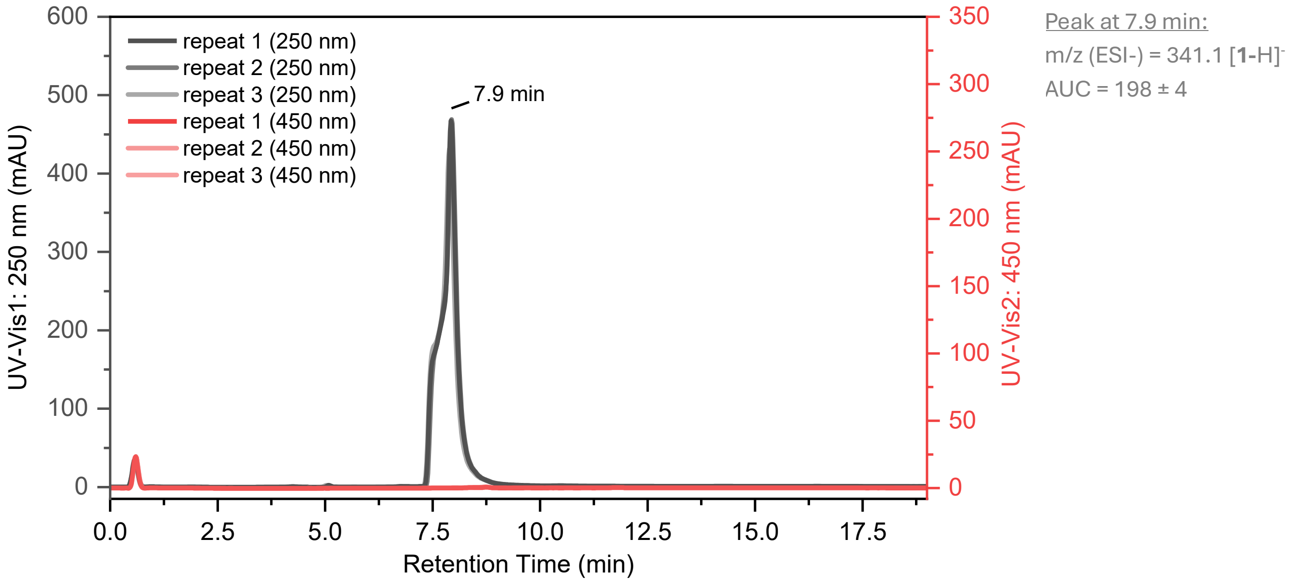

Figure S20. HPLC trace of the reaction mixture of **1** (5 mM) in DMSO in the presence of 10 equivalents $TBANO_3$ after 2.5 h at 80°C under open air and light, showing the absorbance at 250 nm (black traces) and 450 nm (red traces). On the right is shown the dominant *m/z* value for the peaks observed in the HPLC traces, as well as their 'area under the curve' (AUC) in mAU*min if appropriate.

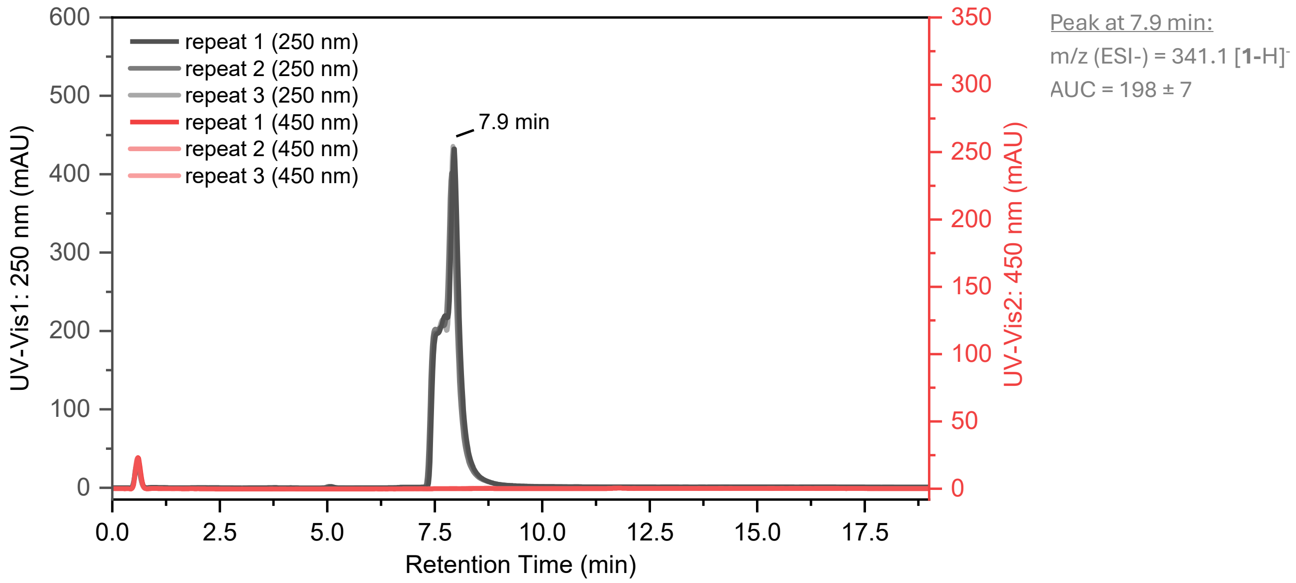

Figure S21. HPLC trace of the reaction mixture of **1** (5 mM) in DMSO in the presence of 10 equivalents $TBAH_2PO_4$ after 2.5 h at 80°C under open air and light, showing the absorbance at 250 nm (black traces) and 450 nm (red traces). On the right is shown the dominant *m/z* value for the peaks observed in the HPLC traces, as well as their 'area under the curve' (AUC) in mAU*min if appropriate.

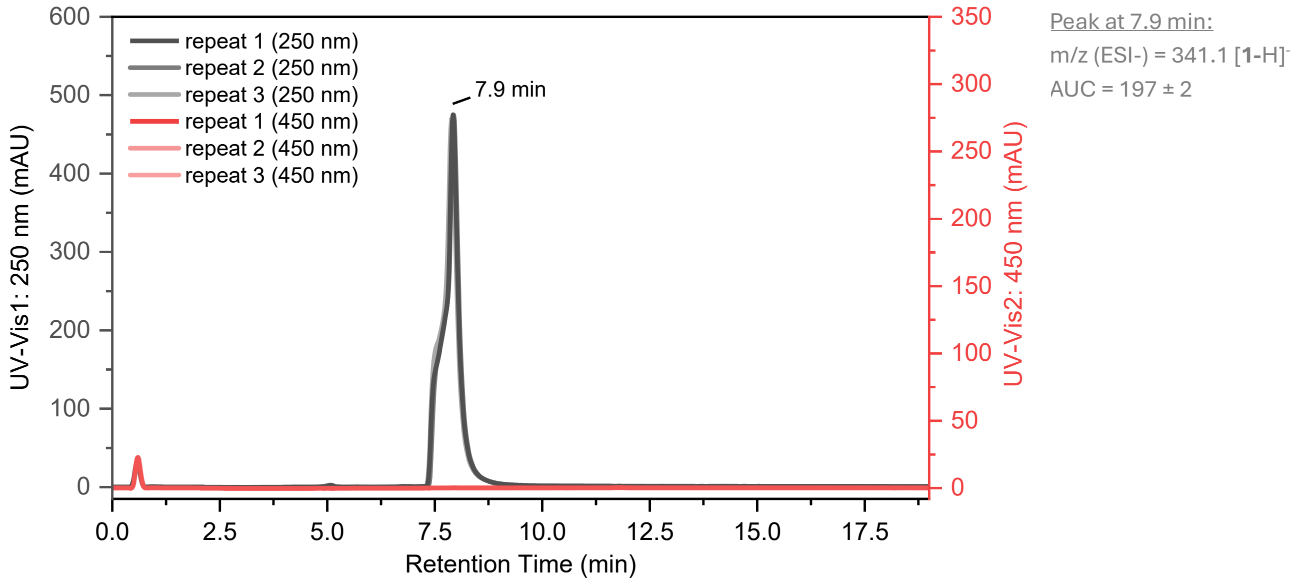

Figure S22. HPLC trace of the reaction mixture of **1** (5 mM) in DMSO in the presence of 10 equivalents TBA_2SO_4 after 2.5 h at 80°C under open air and light, showing the absorbance at 250 nm (black traces) and 450 nm (red traces). On the right is shown the dominant *m/z* value for the peaks observed in the HPLC traces, as well as their 'area under the curve' (AUC) if appropriate.

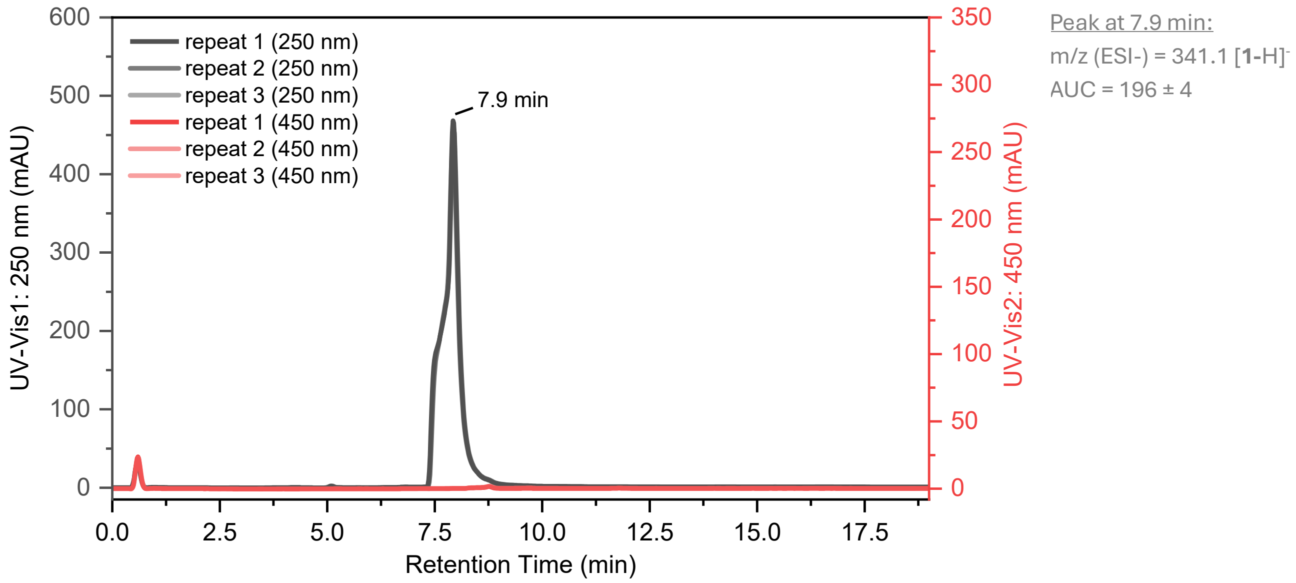

Figure S23. HPLC trace of the reaction mixture of **1** (5 mM) in DMSO in the presence of 10 equivalents $TBAHCO_3$ after 2.5 h at 80°C under open air and light, showing the absorbance at 250 nm (black traces) and 450 nm (red traces). On the right is shown the dominant *m/z* value for the peaks observed in the HPLC traces, as well as their 'area under the curve' (AUC) in mAU*min if appropriate.

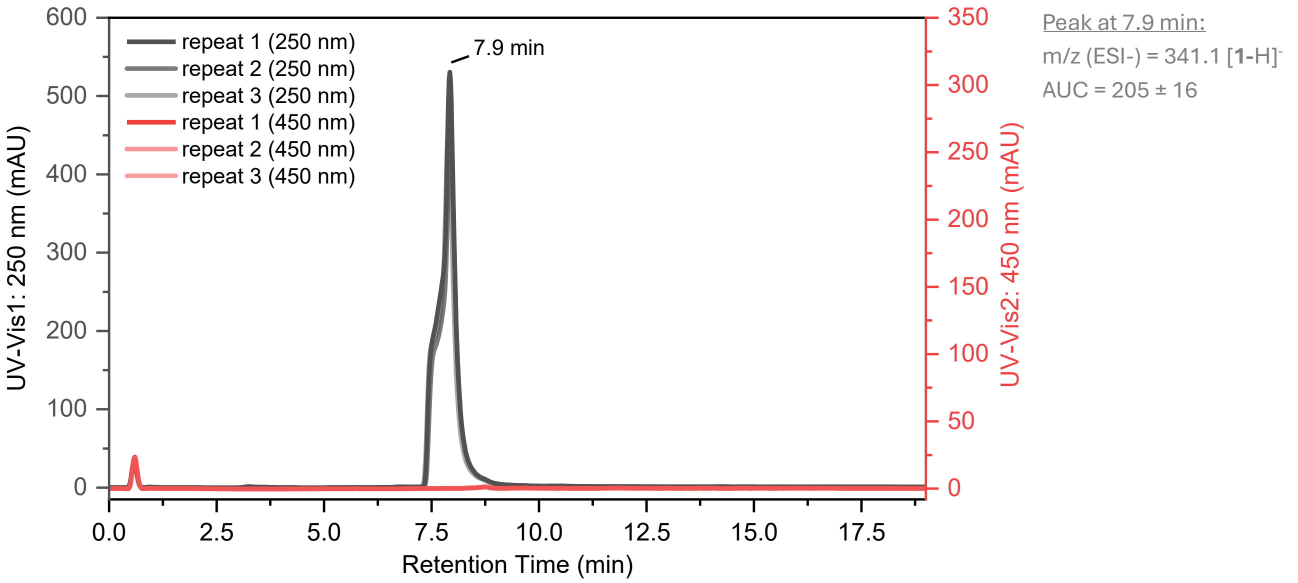

Figure S24. HPLC trace of the reaction mixture of **1** (5 mM) in DMSO in the presence of 10 equivalents triethylamine after 2.5 h at 80°C under open air and light, showing the absorbance at 250 nm (black traces) and 450 nm (red traces). On the right is shown the dominant *m/z* value for the peaks observed in the HPLC traces, as well as their 'area under the curve' (AUC) in mAU*min if appropriate.

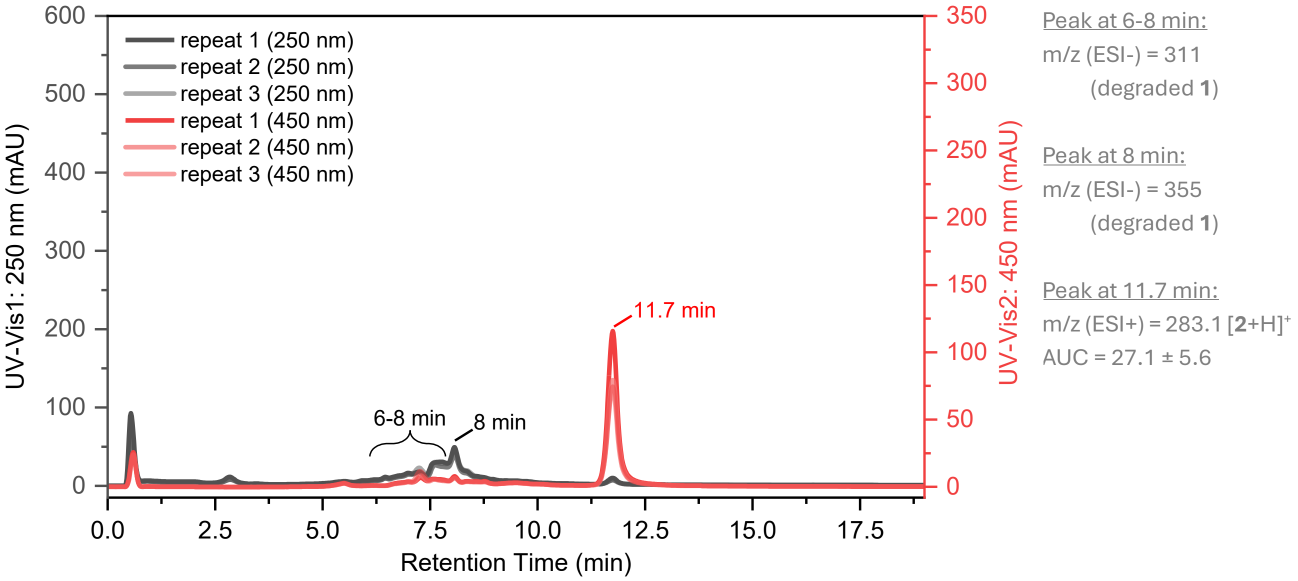

Figure S25. HPLC trace of the reaction mixture of **1** (5 mM) in DMSO in the presence of 10 equivalents DBU after 2.5 h at 80°C under open air and light, showing the absorbance at 250 nm (black traces) and 450 nm (red traces). On the right is shown the dominant *m/z* value for the peaks observed in the HPLC traces, as well as their 'area under the curve' (AUC) in mAU*min if appropriate.

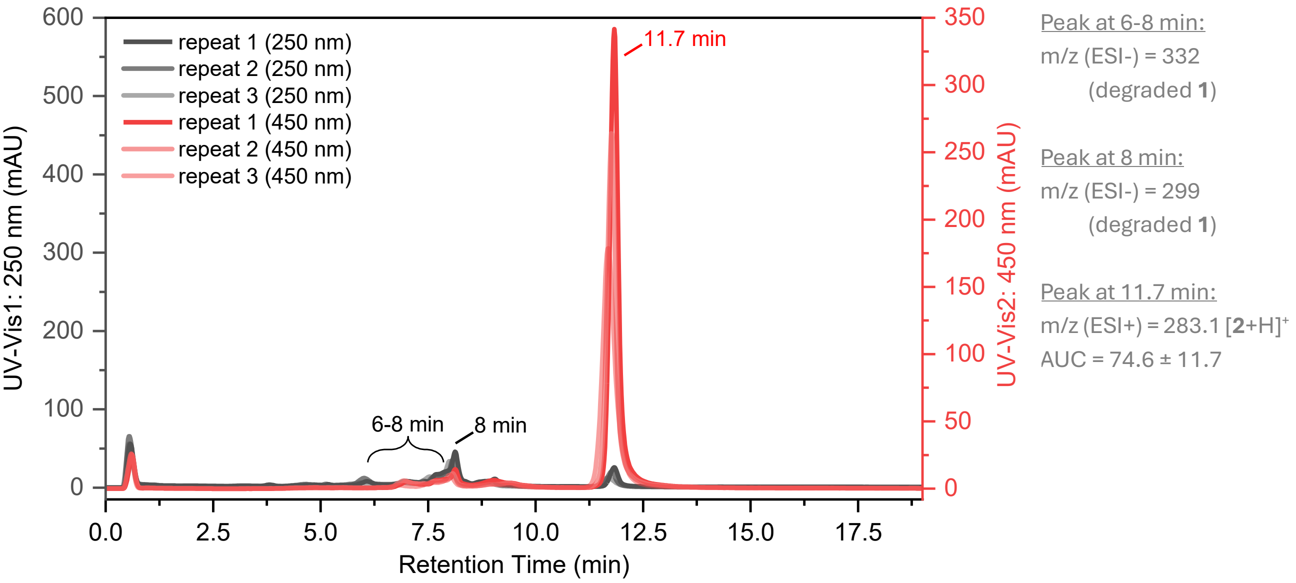

Figure S26. HPLC trace of the reaction mixture of **1** (5 mM) in DMSO in the presence of 10 equivalents TBAOH after 2.5 h at 80°C under open air and light, showing the absorbance at 250 nm (black traces) and 450 nm (red traces). On the right is shown the dominant *m/z* value for the peaks observed in the HPLC traces, as well as their 'area under the curve' (AUC) in mAU*min if appropriate.

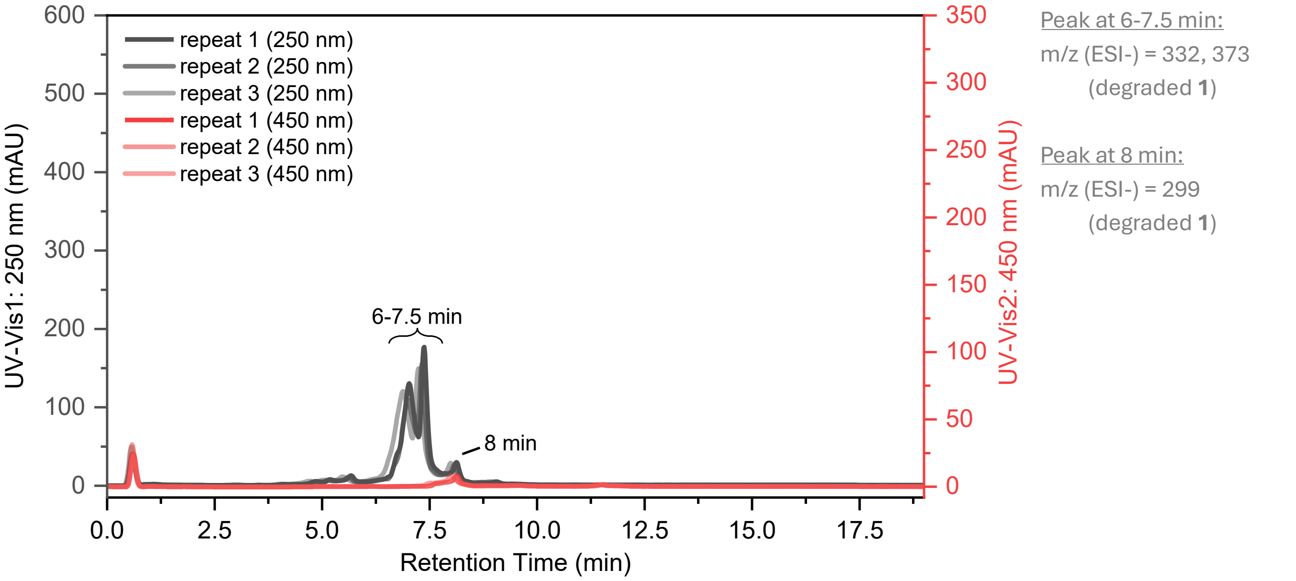

Figure S27. HPLC trace of the reaction mixture of **1** (5 mM) in DMSO in the presence of 10 equivalents TBAOH after 2.5 h at room temperature under open air and light, showing the absorbance at 250 nm (black traces) and 450 nm (red traces). On the right is shown the dominant *m/z* value for the peaks observed in the HPLC traces, as well as their 'area under the curve' (AUC) in mAU*min if appropriate.

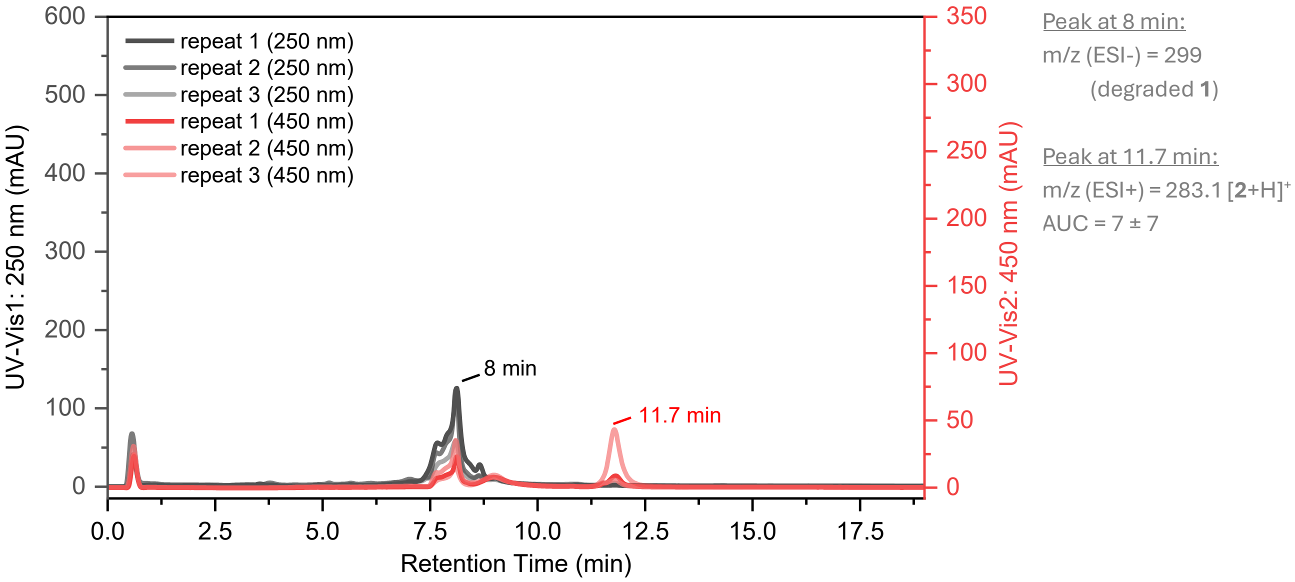

Figure S28. HPLC trace of the reaction mixture of **1** (5 mM) in DMSO in the presence of 10 equivalents TBAOH after 2.5 h at 80°C under argon atmosphere and light, showing the absorbance at 250 nm (black traces) and 450 nm (red traces). On the right is shown the dominant *m/z* value for the peaks observed in the HPLC traces, as well as their 'area under the curve' (AUC) in mAU*min if appropriate.

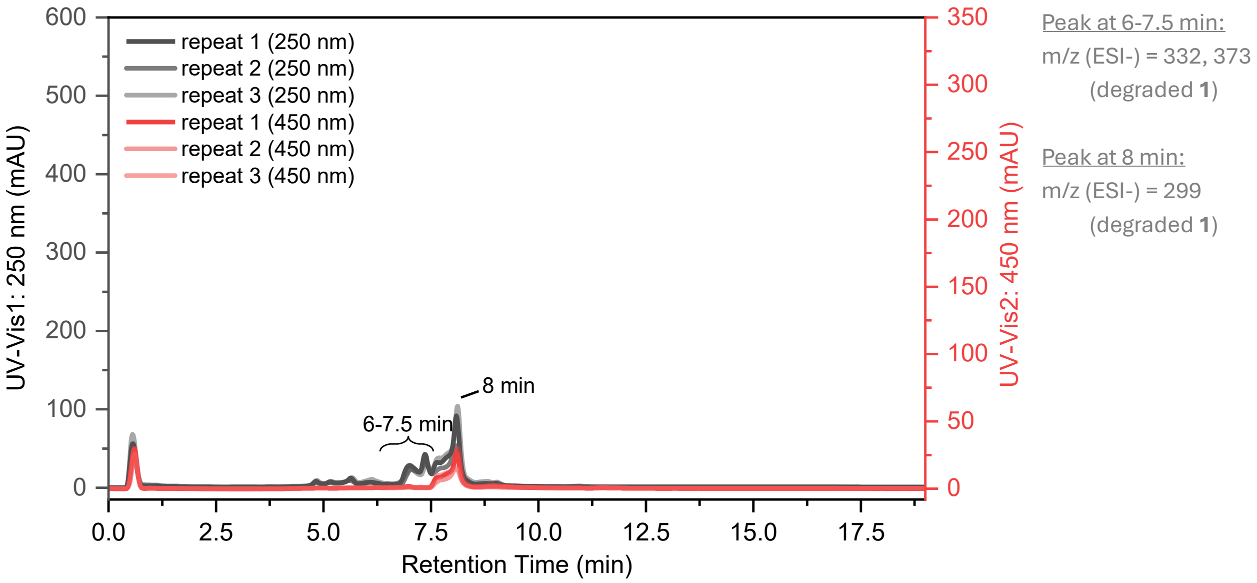

Figure S29. HPLC trace of the reaction mixture of **1** (5 mM) in DMSO in the presence of 10 equivalents TBAOH after 2.5 h at 80°C under open air and kept in the dark, showing the absorbance at 250 nm (black traces) and 450 nm (red traces). On the right is shown the dominant *m/z* value for the peaks observed in the HPLC traces, as well as their 'area under the curve' (AUC) in mAU*min if appropriate.
